# Turmeric‐Derived Nanoparticles Functionalized Aerogel Regulates Multicellular Networks to Promote Diabetic Wound Healing

**DOI:** 10.1002/advs.202307630

**Published:** 2024-03-05

**Authors:** Bodeng Wu, Weilun Pan, Shihua Luo, Xiangrong Luo, Yitao Zhao, Qi Xiu, Mingzhen Zhong, Zhenxun Wang, Tong Liao, Ningcen Li, Chunchen Liu, Chengtao Nie, Guanghui Yi, Shan Lin, MengChen Zou, Bo Li, Lei Zheng

**Affiliations:** ^1^ Department of Laboratory Medicine Nanfang Hospital Southern Medical University Guangzhou 510515 China; ^2^ Center for Clinical Laboratory Diagnosis and Research Affiliated Hospital of Youjiang Medical University for Nationalities Baise 533000 China; ^3^ Key Laboratory of Research on Clinical Molecular Diagnosis for High Incidence Diseases in Western Guangxi of Guangxi Higher Education Institutions Affiliated Hospital of Youjiang Medical University for Nationalities Baise 533000 China; ^4^ Department of Endocrinology and Metabolism Nanfang Hospital Southern Medical University Guangzhou 510515 China; ^5^ Department of Joint Surgery and Sports Medicine The Third Affiliated Hospital of Southern Medical University Guangzhou 510630 China

**Keywords:** aerogels, cross‐kingdom regulations, diabetic wound healing, Nrf2/HO‐1 pathway, turmeric‐derived nanoparticles

## Abstract

Regulation of excessive inflammation and impaired cell proliferation is crucial for healing diabetic wounds. Although plant‐to‐mammalian regulation offers effective approaches for chronic wound management, the development of a potent plant‐based therapeutic presents challenges. This study aims to validate the efficacy of turmeric‐derived nanoparticles (TDNPs) loaded with natural bioactive compounds. TDNPs can alleviate oxidative stress, promote fibroblast proliferation and migration, and reprogram macrophage polarization. Restoration of the fibroblast–macrophage communication network by TDNPs stimulates cellular regeneration, in turn enhancing diabetic wound healing. To address diabetic wound management, TDNPs are loaded in an ultralight‐weight, high swelling ratio, breathable aerogel (AG) constructed with cellulose nanofibers and sodium alginate backbones to obtain TDNPs@AG (TAG). TAG features wound shape‐customized accessibility, water‐adaptable tissue adhesiveness, and capacity for sustained release of TDNPs, exhibiting outstanding performance in facilitating in vivo diabetic wound healing. This study highlights the potential of TDNPs in regenerative medicine and their applicability as a promising solution for wound healing in clinical settings.

## Graphical abstract

TDNPs@AG (TAG), an aerogel dressing containing turmeric‐derived nanoparticles (TDNPs), enhances diabetic wound healing by continued controlled release of TDNPs, which reduce inflammation and oxidative stress. Owing to their bioregulatory properties, water–air balance, easy preparation, and storage capacity, TAG holds promise for tissue regeneration.

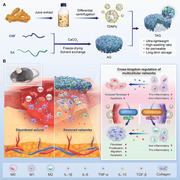

## 1 Introduction

Diabetes is a chronic metabolic disorder affecting 536.6 million people worldwide, and its prevalence is expected to increase to 783.2 million by 2045.^[^ ^1^ ^]^ About 25% of patients with diabetes experience delayed and non‐healing wounds, resulting in prolonged pain, reduced vitality, and even amputation of the lower limbs.^[^ ^2^, ^3^, ^4^ ^]^ Chronic diabetic wounds are caused by a disordered multicellular regulatory network, including long‐term inflammation, inhibited cell proliferation, and micro‐circulatory dysfunction.^[^ ^5^, ^6^ ^]^ It is well understood that ameliorating the wound microenvironment and cell–cell communication benefits diabetic wound healing.^[^ ^7^, ^8^ ^]^ Conservative clinical treatments such as wound debridement, infection control, and inflammation reduction have limited benefits and can exhibit adverse side effects in some patients.^[^ ^9^, ^10^ ^]^ Thus, these mono‐functional therapeutic strategies are inadequate for managing the complex biological processes involved in tissue regeneration.

Extracellular vesicles and particles (EVPs) are nanosized vesicles that can contain a variety of parental molecules such as proteins, nucleic acids, lipids, and other bioactive cargos that act as messengers in cellular communication.^[^ ^11^ ^]^ Due to their diverse content, EVPs exhibit promising potential for providing comprehensive therapeutic efficacy in tissue engineering.^[^ ^12^, ^13^ ^]^ For instance, EVPs secreted by adipose‐derived, epidermal, and mesenchymal stem cells enable the regulation of multiple cellular pathways, thereby accelerating wound repair.^[^ ^14^, ^15^ ^]^ However, the clinical transformation of mammalian‐derived EVPs is limited by low yields and high production costs.^[^ ^12^, ^16^ ^]^

Plant‐derived EVPs, or plant‐derived nanoparticles (PDNPs) are structurally similar to mammalian EVPs,^[^ ^17^ ^]^ but provide several advantages over them—such as abundant sources, easy‐to‐scale production, and low immunological risks, making them attractive alternatives in regenerative medicine.^[^ ^18^ ^]^ Recent studies have shown that PDNPs can affect the biological functions of mammalian cells, thus providing a possible cross‐kingdom regulatory strategy.^[^ ^19^, ^20^ ^]^ Till now, PDNPs derived from grapes, tea, grapefruit, ginseng, ginger, and turmeric have reportedly been effective in the treatment of colitis, liver injury, infection, and tumors.^[^ ^21^, ^22^ ^]^ However, the application of PDNPs to chronic diabetic wound healing is rare and requires further exploration.

Practically, in diabetic wound management, an appropriate strategy for drug administration could promote wound healing and minimize the frequency of interventions required.^[^ ^23^, ^24^ ^]^ As a preferred alternative to solutions, drug‐encapsulated dressings address limitations seen in common wound dressings like medical cotton, bandages, and gauze, which have restricted drug‐loading efficiency and lack tissue adhesion.^[^ ^25^ ^]^ While hydrogels and ointments offer a moist environment, they fall short in breathability and optimal wound exudate management.^[^ ^26^ ^]^ Hence, the key to the transformation of TDNP application to clinical settings lies in the development of a sustained‐release dressing with high drug‐loading capacity, breathability, and tissue adhesiveness. Additionally, extending the storage time and minimizing storage requirements for TDNPs (generally stored at −80 °C) is also crucial.^[^ ^27^, ^28^ ^]^ In this context, aerogels have emerged as promising carriers for TDNPs because of their high drug‐loading capacity, breathability, ultralight weight, and convenient portability and storage.^[^ ^29^, ^30^ ^]^ However, the application of aerogel dressings in wound management is limited by their unknown biocompatibility and complex manufacturing process.

**Scheme 1:**
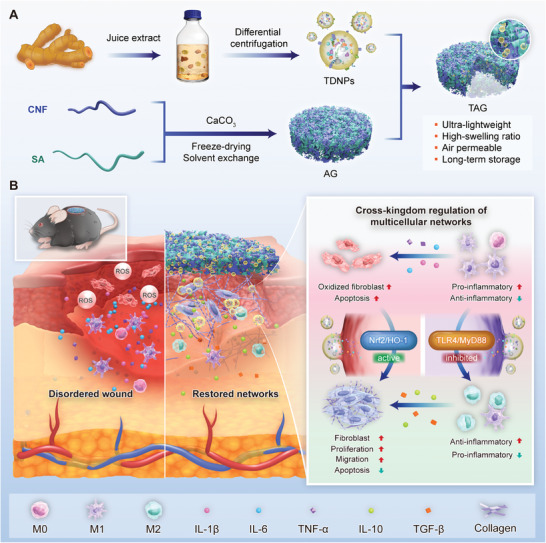
TDNPs‐loaded aerogel serves as an immunomodulatory dressing by modulating multicellular networks for diabetic wound healing. A) Isolation of TDNPs and fabrication of AG and TAG dressings. B) TAG promotes diabetic wound healing by enhancing antioxidant capacity, inhibiting inflammation, and restoring multicellular regulatory networks in the wound microenvironment. TDNPs, turmeric‐derived nanoparticles; AG, aerogel; CNF, cellulose nanofiber; SA, sodium alginate; TAG, TDNPs‐loaded aerogel (TDNPs@AG).

In this study, we analyzed clinical tissue samples and confirmed severe fibroblast damage, inflammatory macrophage infiltration, and extracellular matrix (ECM) disruption in diabetic wounds. Based on these pathological observations and the metabolomics of TDNPs, we speculate that TDNPs could act as a compound medicine for the synergistic regulation of multicellular signaling networks to accelerate diabetic wound repair. We verified that TDNPs could promote proliferation and migration, improve the endogenous antioxidant capacity of fibroblasts, and reduce apoptosis by activating the Nrf2/HO‐1 signaling pathway. TDNPs can also be taken up by macrophages and decrease the production of proinflammatory cytokines by inhibiting the TLR4/MyD88 axis. Besides, it was verified that the TDNPs restored the intracellular communication of the re‐educated macrophages and fibroblasts, facilitating the formation of ECM and skin tissue remodeling. Additionally, we incorporated TDNPs in an ultralightweight and robust aerogel (AG) constructed with cellulose nanofiber (CNF) and sodium alginate (SA) using an ice‐templating method to develop a wound dressing named TDNPs@AG (TAG) to overcome the challenges in practical application and preservation (**Scheme** **1**A). TAG exhibits water‐dependent tissue adhesion to match irregular wound shapes, excellent air permeability, and a continuous TDNPs release pattern. The advantages of TAG were systematically validated in a mouse diabetic wound model (Scheme 1B), highlighting the potential of this dressing for effectively treating diabetic wounds and shedding light on one‐stop research on PDNPs based on clinical difficulties.

## 2 Results and Discussion

### 2.1 Histological Analysis of Chronic Diabetic Wounds

**Figure 1:**
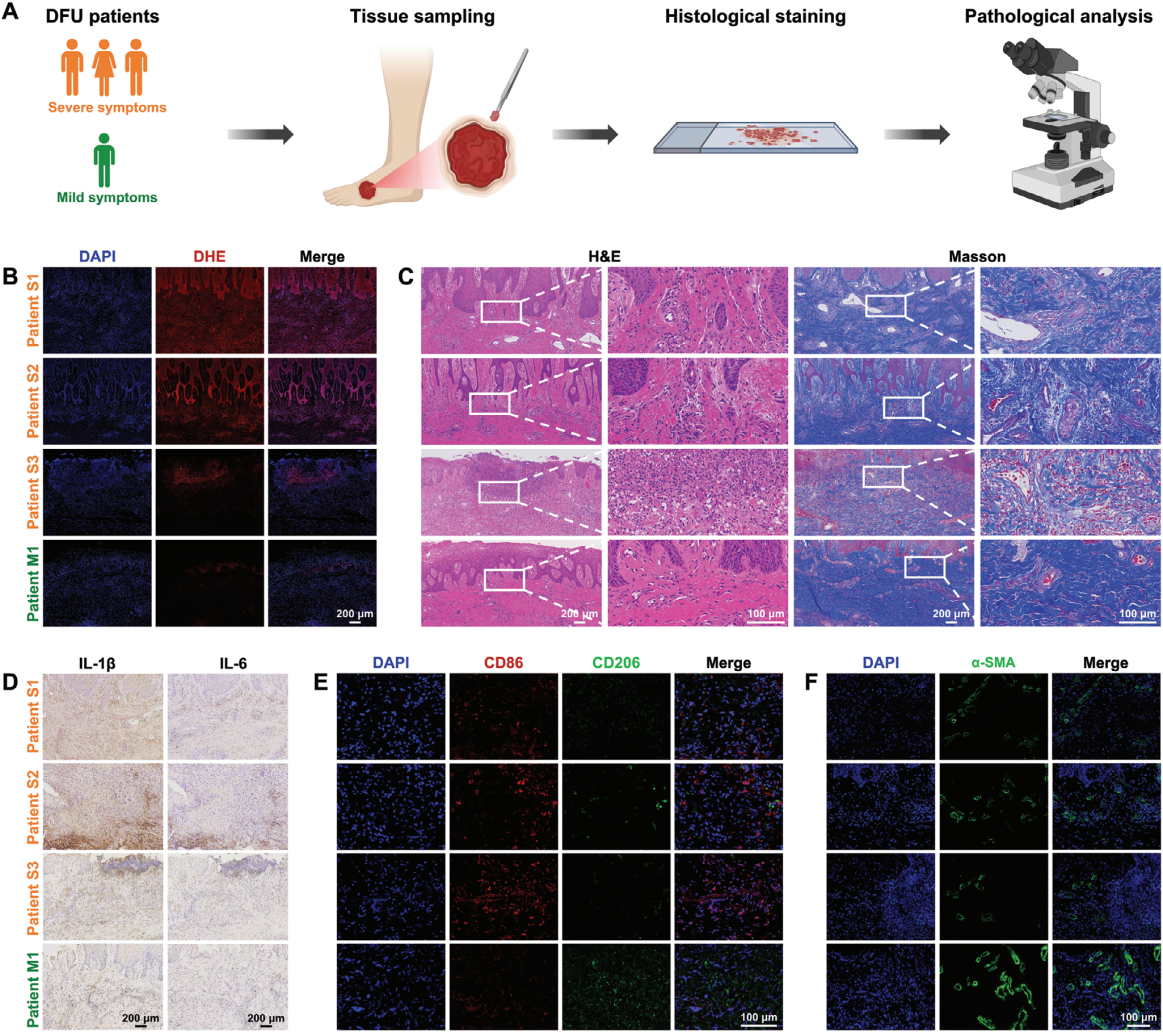
Pathological analysis of DFU tissues from patients with severe (*n* = 3) and mild (*n* = 1) symptoms. A) Flowchart showing collection and analysis of clinical DFU tissues. B) DHE, and C) H&E and Masson's trichrome staining of DFU tissues. D) Expressions of IL‐1β and IL‐6 in DFU tissues. E) Polarization of macrophages in DFU tissues. F) Immunofluorescence staining of α‐SMA in DFU tissues. DFU, diabetic foot ulcer; DHE, dihydroethidium; H&E, hematoxylin and eosin.

To address the current gap between laboratory‐based biomaterial research and its clinical implementation in wound dressing development,^[^ ^31^ ^]^ we initiated the approach of characterizing the histological features of diabetic wounds. Prior to designing PDNP‐based dressings to treat diabetes‐related ulcers, the histological features of diabetic wounds were characterized in the first place. We collected tissue samples from patients with severe or mild symptoms of diabetic foot ulcer (DFU) (**Figure** **1**A), speculating that these differences would shed light on treatment. In this study, three patients with severe DFU (grade 2–4 skin lesions) and one with mild symptoms (grade 0–1 skin lesions) were included according to the Wagner Wound Classification.^[^ ^32^ ^]^

The microenvironment, phenotype, and functionality of cells associated with wound repair in tissue samples were investigated. Dihydroethidium (DHE) staining demonstrated that reactive oxygen species (ROS) significantly accumulated in the tissues of DFU patients with severe symptoms compared to those with mild symptoms (Figure 1B). Hematoxylin and eosin (H&E) staining indicated that inflammatory cells were present in samples from all three patients with severe DFU symptoms. Masson's staining showed that the collagen fibers were disarranged and loose, with gaps in the ECM of the severe DFU symptom samples. In contrast, tissues from DFU patients with milder symptoms showed less inflammatory infiltration with compact and orderly collagen fibers (Figure 1C). Along with this observation, the pro‐inflammatory cytokines of IL‐1β and IL‐6 were also more abundant in the tissues of DFU severe‐symptoms patients (Figure 1D). The M1‐M2 polarization balance of macrophages, a critical immune cell type during DFU recovery, was further verified by immunofluorescence using CD86 and CD206 as markers. CD86^+^ macrophages were more prevalent in tissues from patients with severe DFU symptoms, while the expression of CD206^+^ macrophages was higher in tissues from patients with mild DFU symptoms (Figure 1E). Additionally, the condition of fibroblasts, which are vital cells for wound recovery, was explored. As illustrated in Figure 1F, the expression of fibroblast marker α‐SMA was distinctly reduced in DFU severe‐symptoms patients’ tissues, which might suggest the proliferation and normal metabolism of the fibroblasts were inhibited. Collectively, these findings revealed that poor diabetic wound healing may be caused by an undesirable wound microenvironment with severe inflammation and cell dysfunction. Although our study did not reveal new clinical characteristics of diabetic wounds and was restricted by a small sample size, we observed a correlation between microenvironmental differences and wound severity. Hence, reducing inflammation, and regulating disordered metabolic activity and communication between macrophages and fibroblasts in the DFU wound microenvironment can potentially promote healing of chronic diabetic wounds.

### 2.2 Purification and Characterization of TDNPs

**Figure 2:**
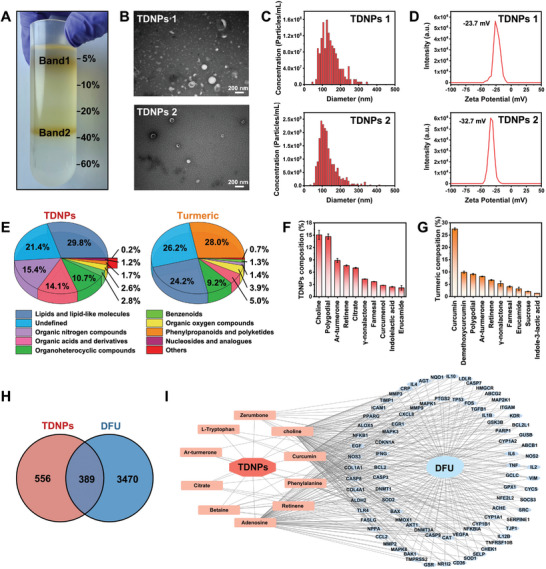
Purification and characterization of TDNPs. A) Two bands formed after iodixanol gradient (5%/10%/20%/40%/60%) ultracentrifugation. B) TEM images of TDNPs 1 and TDNPs 2, respectively. C) Size distributions, and D) zeta potential analysis of TDNPs 1 and TDNPs 2, respectively. E) Pie charts showing the compositions of TDNPs and turmeric tissue (*n* = 3). The top 10 metabolites observed in F) TDNPs and G) turmeric (*n* = 3). H) Potential target gene intersection between TDNPs and DFU. I) Potential target gene network of TDNPs and DFU. Data are presented as mean ± SD. TEM, transmission electron microscope; DFU, diabetic foot ulcer; TDNPs, turmeric‐derived nanoparticles.

Turmeric‐derived nanoparticles (TDNPs) were isolated from homogenized turmeric by differential and density gradient centrifugation. The majority of the TDNPs accumulated at the 5%/10% and 20%/40% interfaces (bands 1 and 2, respectively) of the iodixanol gradient (**Figure** **2**A). The morphology and size of TDNPs from the two bands were characterized using transmission electron microscopy (TEM) and nanoparticle tracking analysis (NTA). TEM results revealed that the product in band 1 (TDNPs 1) was a mixture of lipid vesicles, membrane fragments, and protein‐like particles, whereas the nanoparticles in band 2 (TDNPs 2) exhibited a uniform membrane‐enclosed vesicle structure with relatively low contamination by cell debris and lipoproteins (Figure 2B). In addition, the better yield was calculated in TDNPs 2 (7.56 ± 1.12 mg TDNPs per kg turmeric) compared to TDNPs 1 (2.14 ± 0.75 mg TDNPs per kg turmeric), showing ≈3.5‐fold differences. The size distribution of TDNPs 1 and TDNPs 2 was similar, ranging from 35.0 to 415.0 nm, with a median diameter of 129.5 and 112.5 nm, respectively (Figure 2C). The zeta potential was determined to be −23.7 and −32.7 mV for TDNPs 1 and TDNPs 2, respectively (Figure 2D). Therefore, considering the yield and purity, TDNPs 2 (hereafter referred to as TDNPs in the following text) were selected for subsequent studies.

To investigate compounds that might be pharmacologically beneficial in TDNPs, non‐targeted metabolomics was performed on both TDNPs and turmeric. Total ion chromatograms (TICs) of TDNPs and turmeric were obtained in both negative and positive electrospray ionization modes (Figure S1, Supporting Information), and 211 compounds were identified and annotated (Table S1, Supporting Information). Metabolomic data revealed that both TDNPs and turmeric were abundant in lipids and lipid‐like molecules (29.8% and 24.2%, respectively), organoheterocyclic compounds (10.7% and 9.2%, respectively), and organic oxygen compounds (2.6% and 3.9%, respectively). However, the proportions of organic nitrogen compounds (15.4% vs 1.4%) and organic acids and derivatives (14.1% vs 5.0%) were elevated in the TDNPs (Figure 2E; a detailed comparison is provided in Table S1, Supporting Information). Upon further analysis, we examined the top 10 metabolites with respect to the relative ratio of TDNPs (Figure 2F) to turmeric tissue (Figure 2G). Compared to the top 10 constituents identified in turmeric tissues, TDNPs displayed a marked reduction in curcumin, demethoxycurcumin, and sucrose, whereas the proportions of choline, polygodial, ar‐turmerone, and retinene were significantly increased. These metabolites in TDNPs were involved in the cellular biofunctions of anti‐inflammatory, energy generation, and tissue protection activities, which might play a crucial role in diabetic wound healing.^[^ ^33^, ^34^, ^35^ ^]^ Subsequently, by mining the Comparative Toxicogenomics Database (CTD) and related literature, we selected 10 active compounds in TDNPs for subsequent analysis (Table S2, Supporting Information).^[^ ^36^ ^]^ These active compounds were involved in 945 predicted gene targets, with 389 genes found to intersect between the DFU regulation networks (Figure 2H). After narrowing the focus to genes that had undergone intervention three or more times, 86 potential targets for DFU treatment were identified. The potential drug component‐target‐disease‐pathway network is shown in Figure 2I.^[^ ^37^ ^]^ It was found that the correlated genes were primarily concentrated in pathways linked to inflammation, antioxidation, and apoptosis. Furthermore, pertinent gene enrichment pathways were predominantly associated with diabetic complications, including cell proliferation, apoptosis, and the inflammatory response (Figure S2A,B, Supporting Information). Thus, these results suggest that the enriched bioactive cargos in TDNPs are relevant for regulating DFU cellular pathways, which are expected to exert comprehensive therapeutic efficacy in diabetic wound healing.

### 2.3 TDNPs Promote Proliferation and Migration of Fibroblasts

**Figure 3:**
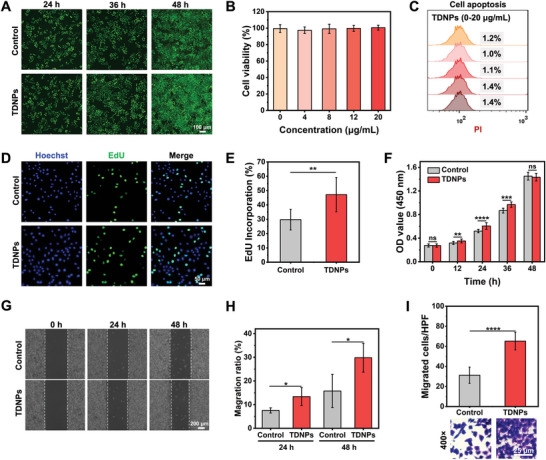
Characterization of TDNPs in fibroblasts. A) Fibroblasts were treated with PBS or TDNPs for different time periods and stained with calcein‐AM and propidium iodide (PI). Green and red fluorescence indicate live and dead cells, respectively. (B) Cell viability of fibroblasts after the addition of TDNPs at varied concentrations (0, 4, 8, 12, and 20 µg mL^−1^, respectively) for 24 h (*n* = 5). C) Analysis of apoptosis of fibroblasts pre‐treated with TDNPs, using PI as an indicator. D) EdU images of fibroblasts treated with PBS and TDNPs. E) Quantification of EdU incorporation (shown in (D)) (*n* = 3). F) Growth trends of fibroblasts with or without treatment with TDNPs over 48 h (*n* = 5). G) Migration capacity of fibroblasts measured using a wound‐healing test. H) Migration ratio of fibroblasts under different formulations (corresponding to(G)) (*n* = 4). I) Statistical analysis and representative images of the migrating cells observed in high power field (HPF), under different formulas (*n* = 3). Data are presented as mean ± SD. Statistical significance was based on Student's *t*‐test and one‐way ANOVA with post‐hoc test; ns, not significant, *p < 0.05, **p < 0.01, ***p < 0.001, and ****p < 0.0001. TDNPs, turmeric‐derived nanoparticles; PBS, phosphate buffer saline; EDU, 5‐ethynyl‐2′‐deoxyuridine.

Considering that the DFU tissue samples demonstrating the metabolism and proliferation of fibroblasts were disordered and thus led to arrested tissue regeneration (Figure 1F), we explored the cross‐species regulation of TDNPs in murine fibroblast L929 cells. Firstly, the biocompatibility of TDNPs was evaluated. It is confirmed that TDNPs did not damage the cell activity (**Figure** **3**A,B) or induce apoptosis (Figure 3C) of fibroblasts with a concentration of up to 20 µg mL^−1^. Considering that the wound‐healing process is highly dependent on cell proliferation and migration, the effect of TDNPs on fibroblasts was investigated. Compared with the cells cultured alone, the proliferation of fibroblasts was enhanced after the addition of TDNPs as suggested by the EdU assay (Figure 3D,E), and thus the TDNPs revealed a continuously growth‐promoting effect on fibroblasts from 12 to 36 h (Figure 3F). Specifically, the proliferation rate at 48 h between TDNPs‐treated cells and controls was comparable, as they reached over 85–90% coverage in culture dishes (Figure 3A). Moreover, the in vitro migration assay indicated that the migration speed of fibroblasts into the scratched areas increased when they were co‐cultured with TDNPs (Figure 3G). Quantitatively, the migration ratio of TDNPs‐treated cells was ≈1.78‐ and 1.89‐fold higher than that of control at 24 and 48 h, respectively (Figure 3H). In addition, the results of the Transwell assay supported this conclusion, with more migrated cells observed in the TDNPs‐treated fibroblast group (Figure 3I). These results confirm that TDNPs positively regulate fibroblast proliferation and migration.

### 2.4 TDNPs Reduce Apoptosis of Fibroblasts by Boosting Endogenous Antioxidant Capacity

**Figure 4:**
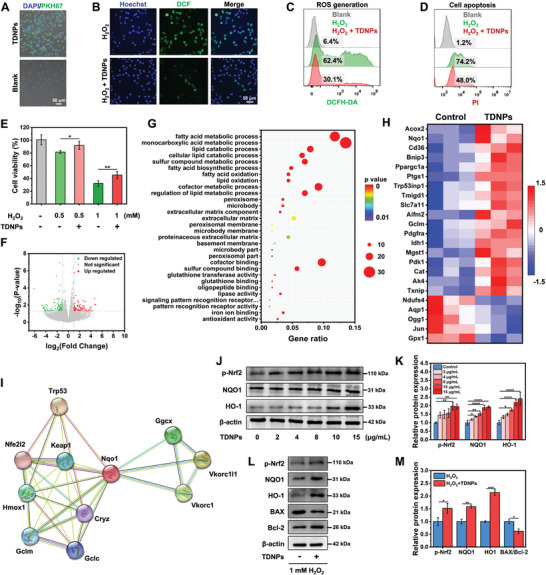
Characterization of TDNPs in fibroblasts. A) Uptake of TDNPs by L929 cells. B) Intracellular ROS level of L929 cells treated with H_2_O_2_ and H_2_O_2_ + TDNPs (*n* = 3). C) Flow cytometry analysis of L929 cells handled with PBS, H_2_O_2_, and H_2_O_2_ + TDNPs, using the DCFH‐DA probe as an indicator (*n* = 3). D) Cell apoptosis of L929 cells after different treatments (*n* = 3). E) Viability of L929 cells under different conditions (*n* = 5). F) Volcano plot analysis of DEGs in L929 cells and TDNPs‐treated L929 cells. G) GO enrichment analysis of the up‐regulated DEGs. H) Upregulated and downregulated genes involved in oxidative stress after TDNPs treatment (fold change ≥ 2 and *p* < 0.05) (*n* = 3). I) Cytoscape plot of STRING protein–protein interaction analysis of Nqo1. J) Western blot analysis of endogenous antioxidant markers p‐Nrf2, NQO1, and HO‐1 in L929 cells after treatment with TDNPs (*n* = 3). K) Quantification of protein expression levels of p‐Nrf2, NQO1, and HO‐1 (corresponding to(J)) (*n* = 3). Values are normalized to that of L929 cells treated with control. L) Western blot analysis of endogenous antioxidant markers of L929 cells under H_2_O_2_ and H_2_O_2_ + TDNPs treatments, respectively (*n* = 3). M) Quantification of protein expression levels of p‐Nrf2, NQO1, HO‐1, and BAX/Bcl‐2 (corresponding to(L)) (*n* = 3). Values are normalized to that of L929 cells treated with control. Data are presented as mean ± SD. Statistical significance was based on Student's *t*‐test and one‐way ANOVA with post‐hoc test; *p < 0.05, **p < 0.01, ***p < 0.001, and ****p < 0. 0001. TDNPs, turmeric‐derived nanoparticles; ROS, reactive oxygen species; PBS, phosphate buffer saline; DCFH‐DA, dichlorodihydrofluorescein diacetate; DEGs, differentially expressed genes; GO, gene ontology; STRING, search tool for recurring instances of neighboring genes.

Excessive ROS production at the wound site is the primary obstacle to diabetic wound healing (Figure 1B).^[^ ^38^ ^]^ Alleviating the superoxidant state in fibroblasts is favorable for reducing apoptosis and promoting proliferation.^[^ ^39^, ^40^ ^]^ Therefore, we investigated the antioxidant effects of the TDNPs in L929 cells. Similar to other EVPs, TDNPs (labeled with the membrane dye PKH67) were internalized by L929 cells within 4 h of incubation (**Figure** **4**A). Subsequently, L929 cells were pre‐treated with TDNPs for 12 h and stimulated with H_2_O_2_ to simulate oxidative stress. Intracellular ROS levels were detected using a dichlorodihydrofluorescein diacetate (DCFH‐DA) probe, which is activated by ROS to emit green fluorescence. As shown in Figure 4B, less fluorescence was observed in the L929 cells supplemented with TDNPs than in the control cells. The flow cytometry results also showed that the proportion of ROS‐excessive cells decreased from 62.4% to 30.1% in the TNDPs group (Figure 4C). Accordingly, the number of apoptotic fibroblasts decreased from 74.2% to 48.0% (Figure 4D). Consequently, the overall viability of L929 cells was rescued by TDNPs, even when exposed to a high concentration of H_2_O_2_ (1 mM) (Figure 4E). To explore the possible intrinsic antioxidant mechanisms of the TDNPs, endogenous antioxidants were tested in L929 cells. Catalase (CAT) and superoxide dismutase (SOD) activities were enhanced in L929 cells after TDNPs treatment in a concentration‐dependent manner (Figure S3, Supporting Information). These results indicated that TDNPs participate in ROS regulation pathways in fibroblasts.

Encouraged by this finding, we identified global transcriptomic changes in fibroblasts in response to TDNPs treatment. RNA sequencing was performed on L929 cells treated with TDNPs and untreated L929 cells as controls. Using a 2.0‐fold cut‐off and Benjamini–Hochberg false discovery rate of < 0.05 threshold for inclusion, we identified 666 genes that were differentially expressed between the control and TDNPs‐treated groups. Volcano plot analysis of differentially expressed genes (DEGs) (*p* < 0.05) revealed that 288 and 378 genes were upregulated and downregulated, respectively, in the TDNPs‐treated L929 cells (Figure 4F). Gene Ontology (GO) enrichment analysis confirmed that the DEGs were involved in several biological processes, including antioxidants, glutathione binding, glutathione transferase, and lipid oxidation (Figure 4G and Figure S4A, Supporting Information). A gene set enrichment analysis (GSEA) demonstrated that a large proportion of the up‐regulated genes were related to cell metabolism, apoptosis, proliferation, antioxidation, etc. (Figure S4B, Supporting Information). We investigated the effects of TDNPs on the expression of genes associated with oxidative stress. As expected, the heatmap showed that 18 of 23 genes were upregulated in TDNPs‐treated L929 cells (*p* < 0.01), and many of these genes were known transcriptional targets of the cellular antioxidant system, including Acox2, Nqo1, Cd36, and CAT (Figure 4H). To further elucidate the role of DEGs related to the cellular oxidation state, we constructed a protein‐protein interaction network using the upregulated gene Nqo1, which plays a vital role in redox regulation. The Search Tool for Recurring Instances of Neighboring Genes (STRING) database indicated that Nqo1 is related to the Nrf2/HO‐1 pathway, which is the principal protective response to oxidative and electrophilic stress (Figure 4I).^[^ ^41^ ^]^ It was also found that the Nrf2/HO‐1 pathway regulates the expression of both CAT and SOD, which work in tandem to maintain adequate levels of intracellular antioxidants.^[^ ^42^, ^43^ ^]^

Therefore, the Nrf2/HO‐1 axis in L929 cells was investigated using western blotting. TDNPs treatment upregulated Nrf2 (p‐Nrf2) phosphorylation. Accordingly, the expression of NQO1 and HO‐1 significantly increased, which is considered effective in balancing oxidative conditions. These changes were observed in L929 cells in a concentration‐dependent manner (Figure 4J,K). Additionally, TDNPs increased the expression of p‐Nrf2, HO‐1, and NQO1 and decreased the ratio of BAX/Bcl‐2 in L929 cells under oxidative stress conditions (Figure 4L,M). These alterations indicate that TDNPs could strengthen the intracellular oxidant and anti‐apoptotic abilities of L929 cells, consistent with the results shown in Figure 4B–E. These results demonstrated that TDNPs protected L929 cells against excessive ROS by activating endogenous antioxidant pathways, which is beneficial for diabetic wound healing.

### 2.5 TDNPs Regulate Macrophage Polarization

**Figure 5:**
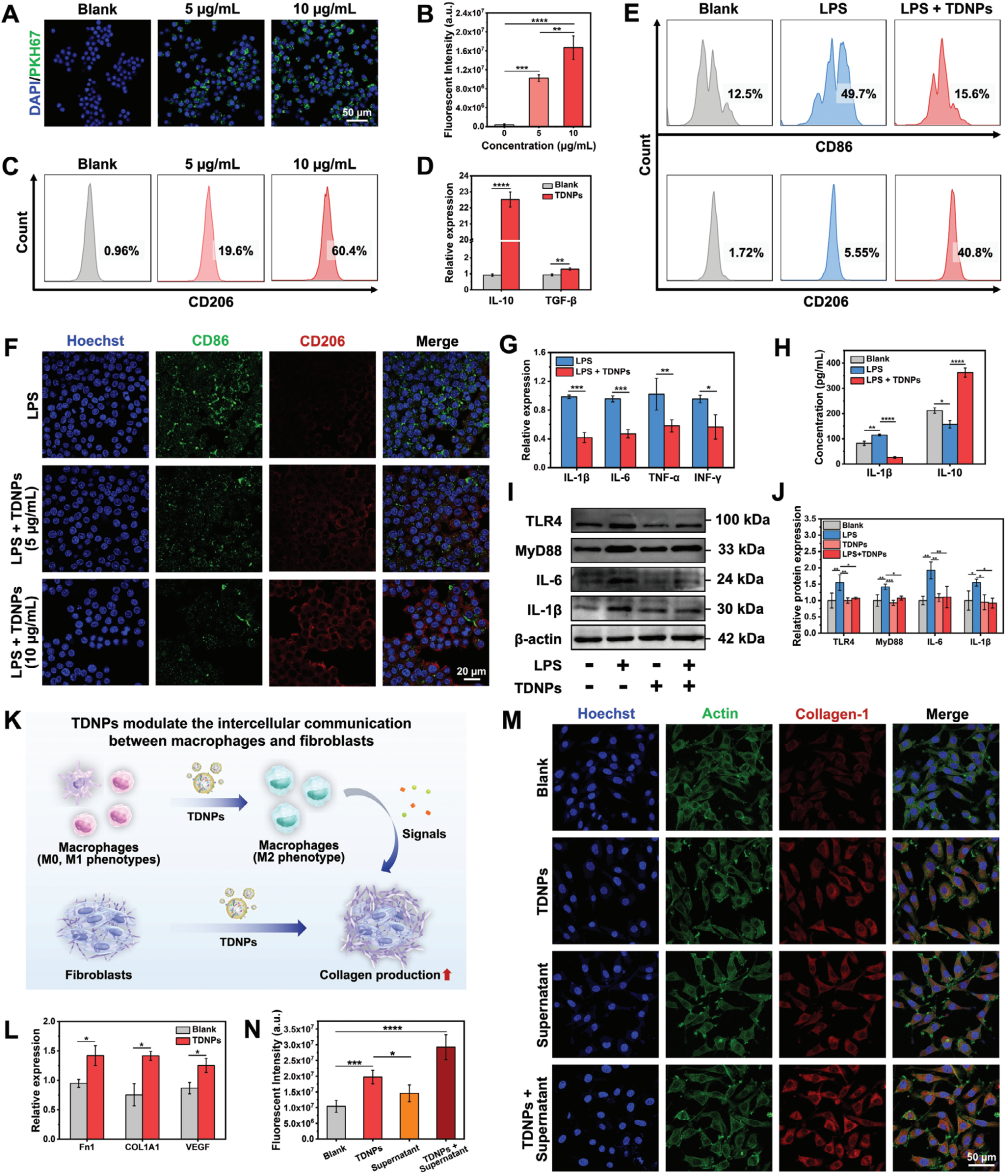
Effect of TDNPs on macrophage polarization and macrophage–fibroblast communication. A) CLSM showing the uptake of TDNPs (green fluorescence) by macrophages. B) Fluorescence intensity analysis of TDNPs ingestion by macrophages (*n* = 3). C) Polarization of macrophages after treatment with TDNPs (5 and 10 µg mL^−1^) (*n* = 3). D) Relative expression of IL‐10 and TGF‐β in macrophages before and after treatment with TDNPs (*n* = 3). E) Polarization of macrophages after treatment with various formulations (*n* = 3). F) CLSM images of macrophages under varied treatments and labeled with CD86 and CD206 fluorescent antibodies. G,H) Expression of cytokines in macrophages assessed by G) qPCR and H) ELISA. (*n* = 3). I) Western blot analysis of TLR4–MyD88 pathway regulated by TDNPs in macrophage (*n* = 3). J) Quantification of protein expression levels of TLR4, MyD88, IL‐6, and IL‐1β (corresponding to(I)) (*n* = 3). Values are normalized to that of L929 cells treated with control. K) Schematic illustration of the influence of TDNPs on communication between macrophages and fibroblasts. L) Gene expressions of Fn1, COL1A1, and VEGF in fibroblasts supplemented with cell supernatant from TDNPs‐treated macrophages (*n* = 3). M) CLSM images of collagen in fibroblasts after various treatments. N) Quantification of fluorescence intensity of collagen (corresponding to(L)) (*n* = 3). Data are expressed as mean ± SD. Statistical significance was based on Student's *t*‐test and one‐way ANOVA with post‐hoc test; *p < 0.05, **p < 0.01, ***p < 0.001, and ****p < 0.0001. TDNPs, turmeric‐derived nanoparticles; CLSM, confocal laser scanning microscopy; LPS, Lipopolysaccharide.

Diabetic wounds suffer from long‐term inflammation, in which resident macrophages are usually polarized into the M1 phenotype and release inflammatory factors. It is vital to regulate macrophage polarization at the wound site to control tissue regeneration by secreting multiple cytokines to adjust the functions of surrounding cells and facilitate the construction of the ECM.^[^ ^8^ ^]^ Motivated by metabolomics (Figure 2), the influence of TDNPs on macrophage polarization was investigated. First, TDNPs were stained with the membrane dye PKH67 and co‐cultured with murine macrophages RAW 264.7 cells. Confocal laser scanning microscopy (CLSM) images revealed that TDNPs could also be taken up by RAW 264.7, and the ingestion of TDNPs was related to the input concentrations (**Figure** **5**A,B). After 24 h incubation, the RAW 264.7 cells were polarized into M2‐type macrophages with the marker CD206 increased from 0.96% to 19.6% (5 µg mL^−1^) and 60.4% (10 µg mL^−1^) (Figure 5C). Accordingly, the expression of cytokines IL‐10 and TGF‐β were both increased (Figure 5D), which were considered strong anti‐inflammation factors. To simulate the residential macrophages in inflammatory wounds, RAW264.7, cells were induced to M1‐type by lipopolysaccharide (LPS), after which the cells were treated with TDNPs. As shown in Figure 5E, the proportion of CD86^+^ macrophages (M1‐type) increased to 49.7% in the LPS group compared to that in the control group (12.5%), indicating successful M1 polarization. After the addition of TDNPs, the percentage of M1 macrophages decreased to 15.6%. Meanwhile, the proportion of M2 macrophages increased from 5.55% to 40.8%, implying that TDNPs could re‐educate M1 macrophages. The CLSM images also showed that the number of M1 macrophages (CD86^+^) decreased after treatment with TDNPs, whereas the proportion of M2 macrophages (CD206^+^) significantly increased in a dose‐dependent manner (Figure 5F). Following incubation with TDNPs, the expression of inflammatory cytokines IL‐1β, IL‐6, TNF‐α, and INF‐γ were significantly down‐regulated (Figure 5G). The expression of cytokines, IL‐1β and IL‐10, were both evaluated (Figure 5H), suggesting that TDNPs could be used as a regulator to adjust inflammatory responses. In addition, the TLR4 and IL‐1β genes were found related to the genes affected by TDNPs (Figure 2I). Therefore, we investigated the effects of TDNPs on the TLR4–MyD88 pathway in vitro in an LPS‐induced inflammatory response. Western blot analysis revealed significant downregulation of TLR4, MyD88, IL‐6, and IL‐1β expressions in TDNPs‐treated macrophages, compared to the LPS group (Figure 5I,J); consistent results were observed in subsequent immunofluorescence experiments (Figure S5, Supporting Information). These results indicate that the macrophage polarization induced by TDNPs is related to the classic TLR4–MyD88 pathway.

Recent studies have shown that interactions between macrophages and fibroblasts are essential for inflammation and wound repair.^[^ ^44^ ^]^ Therefore, the effect of TDNPs on the communication network between these two cell types was further investigated. We first treated primary macrophages with TDNPs for 24 h, and the cell supernatant was collected to culture L929 fibroblasts (Figure 5K). Interestingly, the cell supernatant promoted the expression of fibronectin 1 (Fn1), collagen 1 (COL1A1), and vascular endothelial growth factor (VEGF), which are essential for the construction of the ECM (Figure 5L). Furthermore, the immunofluorescence staining results showed that TDNPs and the cell supernatant facilitated collagen formation, and the combined usage of TDNPs and the cell supernatant exhibited a potent synergistic effect (Figure 5M,N). These results suggested that TDNPs can regulate macrophage polarization and simultaneously advance the healing process by promoting interactions between macrophages and fibroblasts.

### 2.6 Synthesis and Characterization of TAG

**Figure 6:**
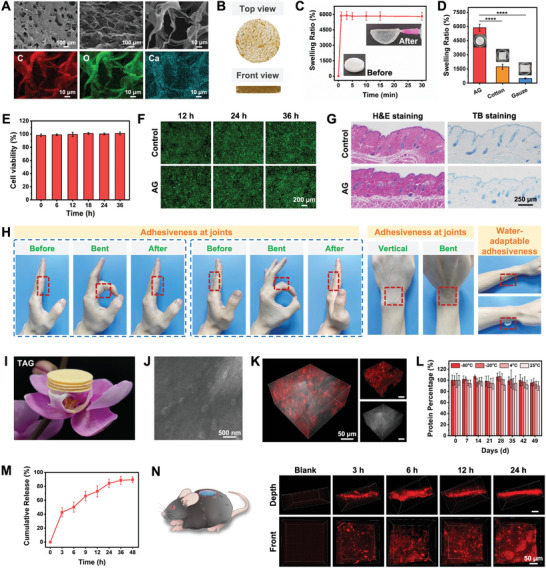
Characterization of TAG. A) SEM image and energy dispersive X‐ray analysis of the aerogel (AG). B) Microtomography of AG showing porous structure. C) Swelling property of AG. The inserts show the AG before and after soaking in water. D) Swelling ratio of AG, medical cotton, and gauze (*n* = 3). E,F) Cytotoxicity of AG in L929 cells (*n* = 5). G) Histological analysis of skin samples collected from mice treated with AG or control (*n* = 3). H) Adhesion of AG on joints and skin surface. I) Picture showing four patches of TAG on a flower. J) SEM image of TAG with TDNPs inside the matrix. K) Distribution of TDNPs within AG. Red fluorescence represents DID‐labeled TDNPs. L) Remaining protein in TAG under different storage conditions for 49 days (*n* = 3). M) Release pattern of TDNPs from TAG (*n* = 3). N) Penetration of TDNPs into the skin tissues. Data are shown as mean ± SD. Statistical significance was based on one‐way ANOVA with post‐hoc test; ****p < 0.0001. TAG, TDNPs‐loaded aerogel (TDNPs@AG); H&E, hematoxylin and eosin; SEM, scanning electron microscope.

Despite the comprehensive therapeutic efficacy of TDNPs in diabetic wound healing, their applicability is limited by stringent storage conditions and inconvenient methods of application. To address the irregularities of diabetic wounds and the need for specialized nursing care, lyophilized TDNPs were incorporated into an aerogel (AG) to obtain TDNPs@AG (TAG). The AG scaffold was synthesized from cellulose nanofibers (CNF), sodium alginate (SA), and calcium carbonate (CaCO_3_) particle suspensions using an ice‐templating method.^[^ ^45^ ^]^ The formation of AG was determined by powder X‐ray diffraction (PXRD), with the characteristic peaks of CNF and alginate shown in the pattern (Figure S6, Supporting Information). SEM revealed the lamellar and porous structure of AG with obvious C, O, and Ca (**Figure** **6**A). Microtomographic analysis also indicated abundant porosity within the AG, with the formation of an interconnected framework (Figure 6B). Owing to its inherently highly porous and low‐density properties, AG presented a swelling ratio of ≈6000%, and the swelling process was saturated within 1 min (Figure 6C, and Figure S7, and Video S1, Supporting Information). This excellent characteristic outperformed the commonly used medical cotton and gauze, showing superior performance in loading TDNPs or absorbing exudates (Figure 6D). The AG also exhibited an outstanding water vapor transmission rate (WVTR) with 1967 ± 193 g (m^2 ^per day)^−1^, fulfilling the general requirement of ≥ 500 g (m^2 ^per day)^−1^, which promises the breathability of the AG when applying on the wound (Table S3, Supporting Information). The shape and thickness of the AG can also be easily modified to accommodate different application scenarios (Figure S8, Supporting Information). Moreover, the tensile strength and compressibility of the synthetic AG could be adjusted by adjusting the concentration of the crosslinking agent (CaCO_3_) to suit diverse applications (Figure S9, Supporting Information).

A series of evaluations was carried out to better understand the biocompatibility of AG. AG was incubated with L929 cells for different time periods and showed no apparent cytotoxicity (Figure 6E,F). The hemolytic assay confirmed that the hemolysis of red blood cells was less than 5% (general requirement) after treatment with AG for 6 h (Figure S10, Supporting Information). Furthermore, to verify the in vivo safety of AG, we attached it to the dorsal skin of C57 mice for four weeks using mice without any treatment as a negative control, and mice administered the sensitizer 1‐chloro‐2,4‐dinitrobenzene (DNCB) as a positive control. As illustrated in Figure 6G and Figure S11, Supporting Information, no hypersensitivity response or mast cell infiltration was observed in the skin tissue of AG‐treated mice, as indicated by H&E and toluidine blue (TB) staining. However, the epidermal layers of DNCB‐treated mice were thicker and massive numbers of mast cells were observed. We further examined the allergic biomarker IgE in the mouse serum and found no negative effects of AG (Figure S12, Supporting Information). Collectively, these results imply that AG can be safely used for biological applications. It is worth noticing that AG exhibited adequate adhesiveness at joints and remarkable skin conformability, which allows it to stick to the wounds even during movement (Video S2, Supporting Information). Intriguingly, the adhesiveness of AG showed a water‐adaptable feature, meaning that AG only gets attached to the skin when wet (Figure 6H). This adaptability enables versatile applications for both AG and TAG, facilitating easy placement on dry wounds with saline solution for moisture or direct application to remove excess exudates in highly‐exuding wounds, thereby promoting an optimal healing environment.

Subsequently, TDNPs were loaded into AG to form TAG dressings using a freeze‐drying approach. As shown in Figure 6I, the TAG possesses an ultra‐lightweight attribute. The morphology of the TDNPs in the TAG was clearly observed by SEM with an intact structure (Figure 6J). To further verify the distribution of TDNPs in the 3D TAG network, the TDNPs were first labeled with the membrane dye DID and loaded onto AG. As shown in Figure 6K, the TDNPs were uniformly distributed in the porous scaffold, showing good dispersibility and high loading capacity. Note that the storage of TDNPs was recommended at −80 °C to retain their bioactivity as much as possible, yet this harsh condition apparently is not suitable for most medical situations.^[^ ^46^ ^]^ Here, by utilizing the AG and freezing‐drying method, the integrity of TDNPs could be maintained under −80 °C, −20 °C, 4 °C, and even 25 °C for 49 days (Figure 6L and Figure S13, Supporting Information), which proposed potential applicability in the clinic.

In addition, the release patterns of TDNPs from TAG were evaluated in vivo. As shown in Figure 6M, TAG presented a burst release behavior (≈40% of TDNPs) at the 3^rd^ hour, and the remaining were released within 48 h with ≈87% cargo of TAG. Nevertheless, studies have shown that it is difficult for large molecules to cross the ECM and be absorbed by skin cells, which reduces their therapeutic effect.^[^ ^47^ ^]^ Therefore, we conducted in vivo uptake experiments. The results demonstrated that the TDNPs (red fluorescence) released from TAG exhibited excellent penetration into wounded skin tissues at a depth of dozens of micrometers (Figure 6N), showing a prominent utilization ratio and potential in vivo cross‐kingdom regulation.

### 2.7 In Vivo Diabetic Wound Healing by TAG

**Figure 7:**
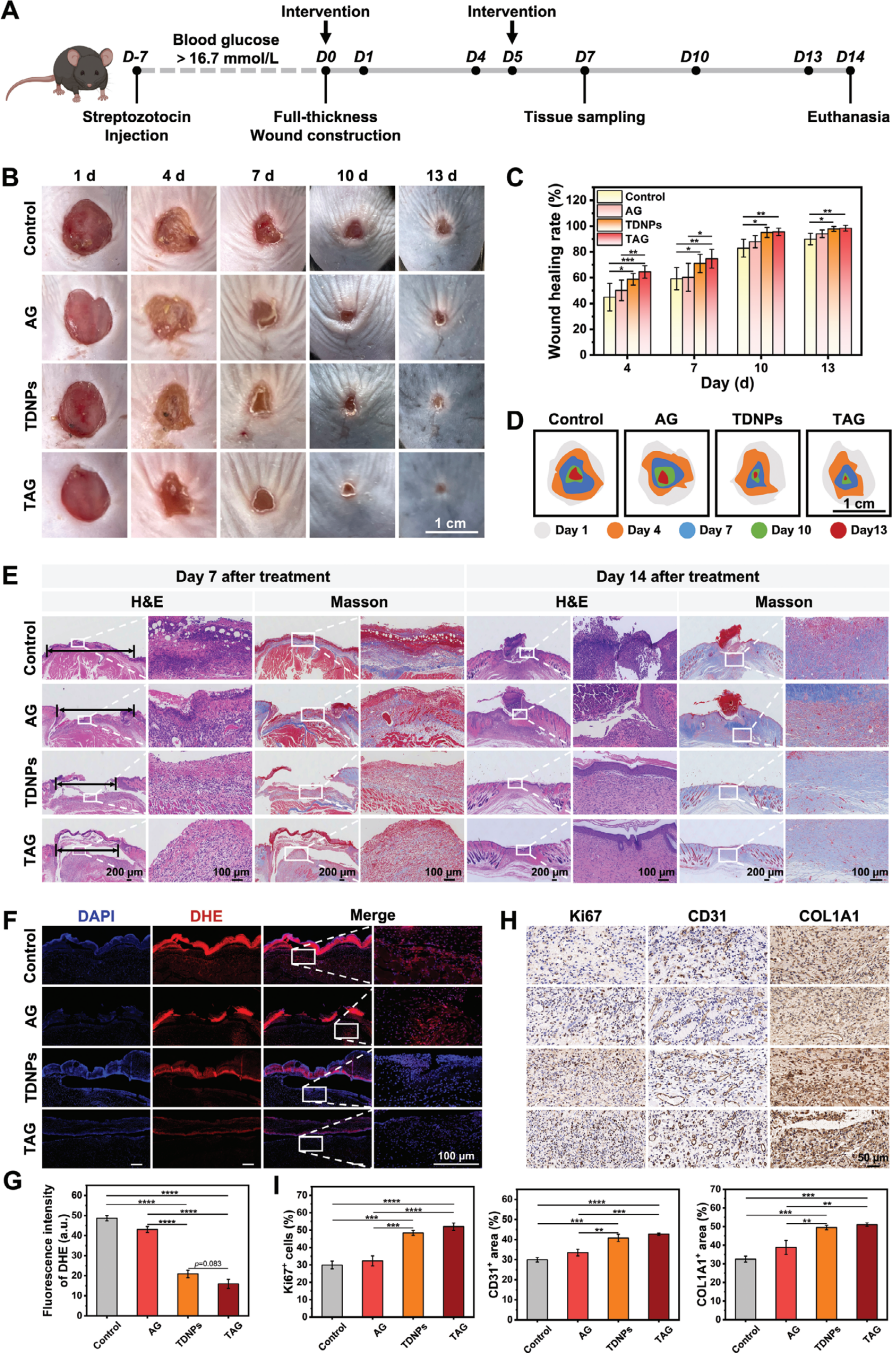
In vivo wound‐healing effect of TAG. A) Flowchart of experimental procedures. B) Representative photographs of diabetic wounds treated with different formulations on days 1, 4, 7, 10, and 13, respectively. C) Wound contractions of mice treated with PBS, AG, TDNPs, or TAG (*n* ≥ 7). D) Schematic images of diabetic wound contraction in mice within 13 days of treatment with different formulations. E) H&E and Masson's trichrome staining of the wound tissues obtained from diabetic mice with varied dressings on days 7 and 14, respectively (*n* = 3). F) Representative DHE staining images of diabetic wounds in each group. G) Statistical data of DHE fluorescence intensity at the wound bed on day 7 (*n* = 3). H) Representative images of Ki67, CD31, and COL1A1 expressions in different groups of tissues. I) Statistical data of Ki67^+^ cells, CD31^+^ area, and COL1A1^+^ area in tissues obtained from different groups (*n* = 3). Data are presented as mean ± SD. Statistical significance was based on one‐way ANOVA with post‐hoc test; *p < 0.05, **p < 0.01, ***p < 0.001, and ****p < 0.0001. TDNPs, turmeric‐derived nanoparticles; TAG, TDNPs‐loaded aerogel (TDNPs@AG); PBS, phosphate buffer saline; AG, aerogel; DHE, dihydroethidium; H&E, hematoxylin and eosin.

The wound‐healing potential of TAG was evaluated in vivo using a full‐thickness skin defect model in diabetic mice (**Figure** **7**A). The mice were divided into four groups and treated with phosphate buffer saline (PBS), AG, TDNPs, or TAG on days 0 and 5 after wound creation. No significant differences were observed in the body weights of any mice throughout the treatment process (Figure S14, Supporting Information). As shown in Figure 7B, the TAG and TDNPs treatment groups substantially accelerated the wound healing process compared to the AG and control groups. In the control group, there was obvious ulceration and exudation on day 1, and redness and thick scabbing on day 4, whereas the TAG group had no obvious redness or swelling. It was observed that the TDNPs and TAG groups showed a higher wound contraction ratio than the other two groups, along the healing process (Figure 7C,D). Although the TAG treatment group did not exhibit a significantly higher healing efficiency than the TDNPs group, it demonstrated a relatively enhanced therapeutic effect during the early stages of wound healing (days 4 and 7). This suggests that sustained release of TDNPs from TAG did not compromise its therapeutic activity, as compared to the application of TDNPs alone. In addition, the AG carrier may also contribute to exudate absorption and provide a balanced water–air microenvironment in the early phases of wound healing.

Multiple examinations were conducted to elucidate the therapeutic microenvironment of wounds. Inflammatory infiltration in the wound tissues was reduced in the groups administered with TAG and TDNPs after 7 days, with diminished wound width and augmented collagen deposition (Figure 7E and Figure S15, Supporting Information). By day 14, the wounds in the TAG and TDNPs groups had completely healed, with an ongoing rearrangement of the microstructures. In contrast, the control and AG groups displayed significant defects and collagen formation was still in the early stages of regeneration. DHE histological staining revealed elevated levels of ROS in the AG and control groups compared with those in the TDNPs and TAG groups (Figure 7F,G). Additionally, the wounds treated with formulations containing TDNPs exhibited enhanced proliferation (Ki67), angiogenesis (CD31), and collagen production (COL1A1) (Figure 7H,I). Notably, compared to the control group, the AG group displayed a slight improvement in wound recovery, collagen formation, and ROS alleviation, which was attributable to the exudate absorption property and moist environment provided by AG. Overall, TAG demonstrated effective cross‐kingdom regulation of natural phytological drugs in mammalian tissues.

**Figure 8:**
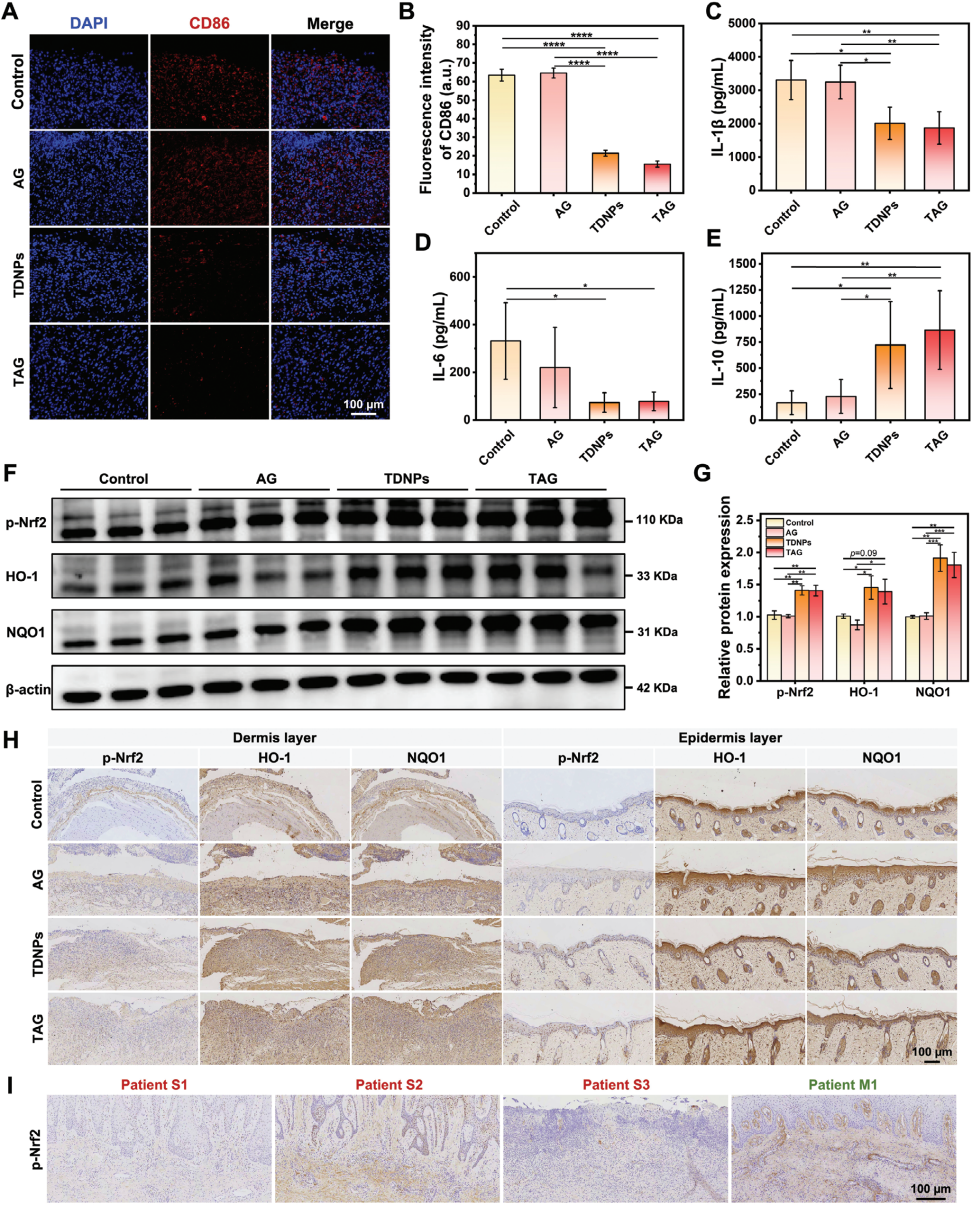
In vivo anti‐inflammatory and antioxidant effect of TAG. A) Representative images of immunofluorescence staining with CD86 in diabetic cutaneous ulcer tissue obtained from mice with varied dressing. B) Quantification of fluorescence intensity (CD86) (*n* = 3). C–E) Statistical analysis of IL‐1β, IL‐6, and IL‐10 in skin tissue, measured using ELISA (*n* = 7). F) Western blot analysis of p‐Nrf2, HO‐1, and NQO1 in wound tissues obtained from mice with varied dressings. G) Quantification of protein expression levels of p‐Nrf2, HO‐1, and NQO1 (corresponding to(F)) (*n* = 3). Values are normalized to that of L929 cells treated with control. H) Representative images of p‐Nrf2, NQO1, and HO‐1 expression wound tissues obtained from mice with varied dressings. I) Expression of p‐Nrf2 in skin tissues obtained from DFU patients. Data are presented as mean ± SD. Statistical significance was based on one‐way ANOVA with post‐hoc test; a non‐parametric Wilcoxon rank‐sum test was used to assess the IL‐1β and IL‐6 ELISA data; *p < 0.05, **p < 0.01, ***p < 0.001, and ****p < 0.0001. TDNPs, turmeric‐derived nanoparticles; AG, aerogel; TAG, TDNPs‐loaded aerogel (TDNPs@AG); ELISA, enzyme‐linked immunosorbent assay.

To gain further insight into the intrinsic mechanism of TAG in improving diabetic wound healing, we assessed inflammatory molecules in the wound microenvironment and their corresponding pathways. The fluorescence intensity of CD86 was significantly lower in the skin tissues from the TDNPs or TAG groups than in the control and AG groups (**Figure** **8**A,B). The comparable fluorescence intensity between the control and AG groups indicated that AG itself did not have an anti‐inflammatory effect. Accordingly, the tissue cytokines of IL‐1β and IL‐6 displayed a reduction tendency in TDNPs and TAG groups in comparison to the rest two groups, where IL‐10 exhibited a reversed trend (Figure 8C‐E). Consistent with the pathway prediction in Figure 4I‐K, western blot results indicated that p‐Nrf2, HO‐1, and NQO1 in the tissues were notably upregulated in the TDNPs and TAG groups (Figure 8F,G). Immunohistochemical staining revealed that the expression of p‐Nrf2, HO‐1, and NQO1 was higher in the dermal tissue of the wound in the TDNPs and TAG groups than in the control and AG groups (Figure 8H and Figure S16, Supporting Information). Moreover, p‐Nrf2 was elevated in the epidermis of the TDNPs and TAG groups, suggesting that TDNPs may activate the Nrf2/HO‐1 pathway in keratinocytes. These results were consistent with observations from clinical tissue samples of patients with DFU, where p‐Nrf2 was generally downregulated in patients with more severe symptoms (Figure 8I). Many treatments, including hyperbaric oxygen therapy, promote wound healing by activating Nrf2/HO‐1 through the activation of the antioxidant capacity.^[^ ^48^, ^49^ ^]^ The above findings indicated that TAG treatment provides an oxidative balanced microenvironment for accelerating diabetic wound healing by a plant‐derived natural vesicular drugs‐mediated multicellular networks regulation. Additionally, our development strategy employed AG as a carrier for TDNPs, ensuring both a moist wound repair environment and sustained release for effective regulation of the wound microenvironment without compromising the biological activity of TDNPs.

## 3 Conclusion

This study presents a novel approach for diabetic wound management by developing turmeric‐derived nanoparticle (TDNPs)‐loaded aerogel dressings (TAG) that target the wound microenvironment. Through pathological analysis of clinical tissue samples, we gained valuable insights into the pathological characteristics of diabetic wounds. Metabolomic analysis and target gene prediction further enhanced our understanding of the therapeutic potential of TDNPs. In vitro experiments demonstrated the efficacy of TDNPs in alleviating inflammation, eliminating ROS, and promoting healing in fibroblasts and macrophages. The developed TAG showed excellent biocompatibility, controlled TDNPs release, and water absorption capacity. Furthermore, in vivo evaluation using a diabetic mouse model confirmed the effectiveness of TAG in facilitating diabetic wound healing, while also addressing the specific challenges associated with it. Our research combines both clinical sample and metabolomic analyses, thereby strengthening the relevance and applicability of our findings. These innovative approaches have the potential to substantially enhance the treatment of diabetic wounds by overcoming the limitations of conventional therapies and biologically based treatments.

## 4 Experimental Section

### Ethics Statement

All animal experiments were approved by the Laboratory Animal Management Committee of Southern Medical University (Permit Number: SMUL2022179). The use of human wound materials was approved by the Ethics Committee of the Nanfang Hospital of Southern Medical University (Permit Number: NFEC‐202012‐K6).

### Materials

Radioimmunoprecipitation assay (RIPA) lysis buffer, bicinchoninic acid assay (BCA assay), protein loading buffer, bovine serum albumin (BSA), protease inhibitor cocktail, coomassile blue, cell count kit‐8 (CCK8) assay, 4% paraformaldehyde, triton‐X100, apoptosis and necrosis detection kit, and EdU cell proliferation kit were purchased from Shanghai Beyotime Biotechnology. Phosphate‐buffered saline (PBS), Dulbecco's modified Eagle's medium (DMEM), 0.25% trypsin‐EDTA, Roswell Park Memorial institute‐1640 (RPMI‐1640), fetal bovine serum (FBS), and penicillin‐streptomycin (10 000 U mL^−1^) were purchased from Thermo Fisher Scientific, Inc. The live/dead fluorescence assay reagent, PKH67 dye, and lipopolysaccharide (LPS) were purchased from Sigma‐Aldrich. The catalase (CAT) and superoxide dismutase (SOD) activity detection kits were obtained from Nanjing Jiancheng Co., Ltd. The 4′,6‐diamidino‐2‐phenylindole (DAPI), 2′,7′‐dichlorofluorescin diacetate (DCFH‐DA) and Hoechst 33 342 were purchased from Beijing Solarbio Life Science Inc.

### Isolation and Purification of TDNPs

Fresh turmeric (purchased from farmers’ markets in Guangxi province) was washed with deionized water, soaked in cold PBS, and homogenized in a blender. The turmeric juice was filtered by 212 *µm* filter nets and subjected to sequentially centrifuged at 1000 g for 20 min, 3000 g for 20 min, 10 000 g for 30 min, and 70 000 g for 1 h to remove large particulates. Subsequently, the supernatant was ultracentrifuged at 135 000 g for 70 min, and the pellet was suspended in PBS to obtain crude TDNP samples. TDNPs were purified using OptiPrep (iodixanol) density gradient medium (5%, 10%, 20%, 40%, and 60%[w/v]), followed by centrifugation at 150 000 g for 2 h. The bands between the 20% and 40% layers were harvested after 135 000 g centrifugation for 70 min and were termed TDNPs. The collected TDNPs were stored at −80 °C for further use.

### Characterization of TDNPs

Transmission electron microscopy (TEM, HITACHI) was used to visualize the morphology of the TDNPs. The particle size and zeta potential of TDNPs were measured using a Zetasizer Nano ZS (Malvern). The protein concentration of the TDNPs was determined using a bicinchoninic acid assay.

### Metabolomics Analysis of TDNPs

Turmeric tissues and TDNPs were frozen in liquid nitrogen and ground into a fine powder using a mortar and pestle. One milliliter of methanol was added to the homogenized solution for metabolite extraction. The mixture was centrifuged for 20 min (14 000 g, 4 °C). The supernatant was dried using a vacuum centrifuge.

The analysis was performed using UHPLC (Vanquish UHPLC, Thermo) coupled to an Orbitrap. Samples were separated by a 2.1 × 100 mm Hypersil gold 1.9 µm column (Thermo). In both the positive and negative electrospray ionization (ESI) modes, the mobile phase contained 0.1% formic acid in water and acetonitrile. The gradient was 0–3 min: 5% B; 3–20 min: 30–85% B; 20–23 min: 85–100% B; 23–25 min: 100% B. The flow rate was 300 µL min^−1^. The ESI source conditions were set as follows: sheath gas (Arb) as 40, aux gas as (Arb) as 10, sweep gas (Arb) as 1, ion transfer tube temperature: 320 °C, vaporizer temperature: 350 °C, positive ion: 3500 V, negative ion: 3000 V. In the MS‐only acquisition, the instrument was set to acquire over an m/z range of 100–1000 Da, the resolution was set at 60 000, and the maximum injection time was set at 100 ms. In auto‐MS/MS acquisition, the instrument was set to acquire over an m/z range of 50–1000 Da, the resolution was set at 15 000, and the maximum time was set at 50 ms, excluding times within 5 s. The raw MS data were converted to MzXML files using ProteoWizard MSConvert before importing them into the freely available XCMS software.

### Cell Culture

Mouse fibroblast L929 and mouse RAW264.7, cell lines were obtained from American Type Culture Collection (ATCC) and maintained in DMEM containing 10% FBS and 100 U mL^−1^ penicillin/streptomycin. Primary murine bone marrow‐derived macrophages (BMDM) were generated from the tibia and femur of C57B/L mice (6 weeks old, female) and cultured in complete RPMI‐1640 containing 20% L929 cell supernatant for 7 days. The study design and protocol were approved by the Ethics Committee of Southern Medical University. All cells were incubated at 37 °C in a humidified atmosphere containing 5% CO_2_.

### In Vitro Cytotoxicity of TDNPs

L929 cells were seeded in 6‐well or 96‐well plates and cultured for 24 h. TDNPs at different concentrations were added to the cells and incubated for 24 or 48 h. The cells were washed with PBS and subjected to live/dead fluorescence assay and CCK8 assay, respectively. The apoptosis of L929 cells after incubation with TDNPs was assessed by Annexin V‐FITC and propidium iodide (PI) dual staining using flow cytometry (BD). The reagents were administered according to the manufacturer's guidelines.

### Cell Proliferation Detected by EdU Assay

L929 cells were seeded in 6‐well plates and cultured overnight. TDNPs (10 µg mL^−1^) were added to the cells and incubated for 24 h. Afterward, the cells were washed with PBS and stained with EdU solution (10 µM) for 2 h. Next, the L929 cells were fixed with 4% paraformaldehyde, treated with Triton X‐100 (0.3%), and incubated with detection reagents for 30 min in the dark. Nucleic acids were stained with Hoechst 33342 for 10 min. Images were captured using fluorescence microscopy (excitation wavelengths for Hoechst 33342 and EdU were 350 and 488 nm, respectively).

### Cell Proliferation Detected by CCK8 Assay

L929 cells were seeded in 96‐well plates and cultured overnight. TDNPs (10 µg mL^−1^) were added to cells and cultured for 12, 24, 36, and 48 h, respectively. L929 cells were washed with PBS and supplemented with DMEM (90 µL) containing CCK8 detection reagent (10 µL). After 2 h incubation at 37 °C, the absorbance at 450 nm was measured by a microplate reader.

### Wound‐Healing Assay

The L929 cells were cultured in 6‐well plates and scratched using a pipette tip. The cells were washed with PBS and supplemented with serum‐free DMEM with or without TDNPs (10 µg mL^−1^) for 48 h. The calculation of the wound healing rate was performed using the formula: Wound healing rate  =  [(wound width at 0 h) ^−^ (wound width at each time point)] / (wound width at 0 h) × 100%. Images of scratches were captured using a microscope (Nikon), and the wound closure distance was analyzed using the ImageJ software (National Institutes of Health, USA).

### Transwell Assay

L929 cells were seeded in 6‐well plates and cultured overnight. The cells were washed and cultured in serum‐free DMEM with TDNPs (10 µg mL^−1^) for 24 h. The cells were then collected and placed in the upper chamber of a 24‐well plate. Transwell chambers (Corning) were used for transwell assays. DMEM supplemented with 10% FBS was added to the lower chamber. L929 cells were further cultured for 36 h and non‐metastatic cells were removed using a cotton swab. The migrated cells were fixed with 4% paraformaldehyde and stained with 0.1% crystal violet.

### Cellular Uptake of TDNPs

TDNPs (200 µg) were labeled with PKH67 dye (1 µL PKH67 + 250 *µ*L Diluent C) for 3 min shield from light. Next, an equal volume of 1% BSA in PBS was added for 5 min and the unbound dye and BSA were removed by ultrafiltration (100 kDa) five times. The labeled TDNPs were added to L929/RAW264.7 cells after 4 h of incubation. The cells were then washed with PBS and stained with DAPI before observation under a laser scanning confocal microscope (CLSM, Nikon).

### Intracellular ROS Detection

The ROS generation in L929 cells was measured using the oxidation‐sensitive fluorescent probe DCFH‐DA. After treatment with TDNPs (10 µg mL^−1^) for 12 h, the cells were washed with PBS and incubated with DCFH‐DA (10 µ*m*ol L^−1^) for 20 min. Subsequently, H_2_O_2_ (1 mM) was added to the cells and incubated for 30 min. Finally, the cells were washed with PBS and analyzed using fluorescence microscopy (Nikon) and flow cytometry (BD).

### Antioxidant Ability of TDNPs

The oxidative stress in L929 cells was induced by H_2_O_2_. When the cell density reached 50%, PBS or TDNPs (10 µg mL^−1^) were added and incubated for 12 h. Subsequently, the L929 cells were washed with PBS and treated with fresh DMEM containing H_2_O_2_ (0.5 and 1 mM) for 4 h. The viability of the treated cells was determined using CCK8 and apoptosis detection assays.

### RNA Sequencing (RNA‐seq) and Bioinformatic Analysis

RNA sequencing was conducted using Novogene (Novogene Biotech Co., Ltd.) to analyze the genetic material of L929 cells. Total RNA was extracted using the RNAeasy Mini Kit (Qiagen) and RNA quality was assessed using the RNA Nano 6000 Assay Kit of the Bioanalyzer 2100 system (Agilent Technologies, CA, USA). Briefly, mRNA was purified from RNA samples using poly T oligo‐attached magnetic beads. Library fragments were purified to select cDNA fragments with a preferred length range of 370–420 bp using the AMPure XP system (Beckman Coulter, Brea, CA, USA). The PCR product was purified (AMPure XP system), and library quality was assessed using an Agilent 2100bioanalyzer. Clustering of the index‐coded samples was performed on a cBot Cluster Generation System using the TruSeq PE Cluster kit v3‐cBot‐HS(Illumina) according to the manufacturer's instructions. After cluster generation, the library preparations were sequenced on an Illumina NovaSeq platform, and 150 bp paired‐end reads were generated. Differential expression analysis was performed using the DESeq2R package (version 1.20.0). DESeq2 provides statistical routines for determining differential expression in digital gene expression data, using a model based on a negative binomial distribution. The resulting *p‐*values were adjusted using Benjamini and Hochberg's approach to control for the false discovery rate. Genes with an adjusted *p*‐value < = 0.05 found by DESeq2 were assigned as differentially expressed. Gene Ontology (GO) enrichment analysis of differentially expressed genes was performed using the cluster Profiler R package, in which the gene length bias was corrected. GO terms with corrected *p*‐values less than 0.05 were considered significantly enriched by differentially expressed genes.

### In Vitro Macrophage Polarization

The murine macrophage RAW 264.7 was cultured in glass dishes for 24 h, after which TDNPs (5 or 10 µg mL^−1^) were introduced for 24 h incubation. The cells were washed with PBS and labeled with CD86‐APC and CD206‐PE antibodies (BioLegend) for flow cytometric analysis. The expression of cytokines (IL‐10 and TGF‐β) was further detected through qPCR assay.

Murine bone marrow cells were harvested from 6–8 weeks old female C57BL/6J mice. Bone marrow was dissociated into single cells and filtered through a 70 µm cell strainer, followed by the treatment of red blood cell (RBC) lysis buffer to remove the RBC. The mixture was centrifuged, and the pellet was cultured in DMEM/FBS containing 20 ng mL^−1^ murine GM‐CSF (Peprotech). After 5 days of cultivation, the adherent cells (BMDM) were harvested. To verify the effect of TDNPs on M1‐type macrophages, BMDM were cultured with LPS (100 ng mL^−1^) for 12 h to induce M1 polarization. TDNPs (5 or 10 µg mL^−1^) were used to incubate with M1 macrophages for another 12 h. Afterward, the cells were collected and stained with CD86‐APC and CD206‐PE antibodies and then were measured by flow cytometry. The expression of cytokines (IL‐1β, IL‐6, TNF‐α, and INF‐γ) was further detected through qPCR assay. CLSM images were obtained using a similar method except that the cells were fixed, permeabilized, and labeled with CD86 and CD206 antibodies (Proteintech), secondary fluorescent antibodies (labeled with FITC and Alexa Fluor 647), and Hoechst 33342 before observation. In Western blot and immunofluorescence experiments, LPS (200 ng mL^−1^) and TDNPs (10 *u*g/ml) were employed as treatment doses.

### Detection of Collagen‐1 in L929 Cells

The L929 cells were cultured on glass dishes for 24 h, followed by the addition of PBS, TDNPs (10 µg mL^−1^), supernatant from RAW264.7 (TDNPs pre‐treated for 24 h), TDNPs + supernatant from RAW 264.7 (TDNPs pre‐treated for 24 h), respectively. After incubation for 24 h, L929 cells were washed and fixed with 4% paraformaldehyde, permeabilized with 0.3% Triton X‐100, and treated with goat serum for 30 min. The cells were then incubated with the collagen‐1 antibody at 4 °C, shielded from light overnight, and the second antibody AF647 was further labeled. The cells were stained with fluorescent phallotoxins and Hoechst 33342 before observation using CLSM.

### Synthesis of AG

The AG was prepared using the ice‐templating method. The CNF solution (2%, 120 mL) and alginate solution (0.6%, 10 mL) were mixed and diluted to 232.4 mL with ultrapure water. Subsequently, the Na_2_CO_3_ solution (0.25 M, 25 mL) was dropped into a CaCl_2_ solution (0.25 M, 25 mL) with vigorous stirring in an ice bath for 10 min to obtain a CaCO_3_ particle suspension. Next, the CaCO_3_ particle suspension (34.16 mL) was injected into the mixture of CNF and alginate with stirring for 5 min. The resulting sample was centrifuged at 800 × g for 5 min to remove the air bubbles. The mixture was poured into polystyrene Petri dishes (60 × 15 mm) and the lid was sealed. The molds were placed at 4 °C for 1 h and transferred to the freezer (^−^20 °C) overnight. The frozen hydrogels were placed in an acetone solution containing 10% acetic acid for 2 h at 25 °C. The gels were soaked in an acetone solution for solvent exchange. All the gels were dried naturally in air to obtain the AG.

### Preparation of TAG

TDNPs (1 mg mL^−1^, 0.2 mL) were introduced into the AG (1.5 × 1.5 × 0.3 cm), after which the TAG was lyophilized. TAG served as the sustained release system of TDNPs for subsequent in vivo studies.

### TDNP Release Pattern from TAG

The release pattern of TDNPs from TAG was determined in vivo. C57BL/6J (6–8 weeks) was anesthetized by an intraperitoneal injection of 2% pentobarbital sodium (50 mg kg^−1^). The dorsal hair of the mice was shaved, and 1 × 1 cm punch biopsy specimens were excised from the dorsum. The wound was treated with lyophilized TAG, and 300 µL of normal saline was added to the TAG. The remaining TNDPs in the TAG were measured by the BCA protein assay at 3, 6, 9, 12, 24, 36, and 48 h. The TDNPs release ratio was calculated as the remaining TAG/total protein in the TAG.

### In Vivo Trafficking of TDNPs

In vivo trafficking of TDNPs was demonstrated using DiD‐labeled TDNPs. TDNPs (100 µg per 100 µL per mouse) were first labeled with a DiD dye (Invitrogen, USA, 1 µL DiD dye + 100 µL PBS). The mixture was ultrafiltered several times to remove unbound dye and then resuspended in PBS. The dorsal skin of the diabetic mice (see more details in the section on “diabetic wound healing evaluation”) was scratched using a blunt blade to induce skin damage. Subsequently, the wounded skin was treated with TAG shielded from light for various time points. As controls, the equivalent DiD dye in PBS was ultrafiltered, and the resulting solution was suspended in PBS to treat the mice. The mice were imaged using a multiphoton laser‐scanning microscope (FV1200MPE, Olympus, Japan).

### RT‐PCR Analysis

Total RNA was extracted from the samples using the TransZol Up Plus RNA Kit (TransGen Biotech, China). The quantity and quality of the RNA were verified using a Nanodrop 2000 (Thermo Fisher Scientific). Total RNA (500 ng) was used for the preparation of cDNAs using the TransScript Uni All‐in‐one First‐Strand Synthesis kit. The amplification was conducted on a Light Cycler 480 device (Roche) using the PerfectStart Green qPCR Super Mix kit. Each reaction system (20 µL) contained 2×PerfectStart Green qPCR Super Mix (10 µL), cDNA template (2 µL), primer (10 µM, 1 µL), and water (6 µL). Nuclease‐free water was substituted for cDNA in negative control samples. The amplification program was performed as follows: pre‐denaturation at 94 °C for 30 s followed by 45 cycles at 94 °C for 5 s and 60 °C for 30 s. Triple replicates were used both for each targeted gene and for the test strains. The primers and amplicons used for real‐time qPCR analysis are listed in Table S4, Supporting Information.

### Immunoblotting

Samples were lysed in a RIPA buffer in the presence of protease and phosphatase inhibitors. Proteins were resolved on 5–15% SDS‐polyacrylamide gels and transferred to PVDF membranes. After blocking with 5% BSA, membranes were incubated overnight at 4 °C with the appropriate primary antibodies. After washing with TBS‐0.1% tween‐20 for 5 min, the membranes were soaked in horseradish peroxidase‐conjugated secondary antibodies (1:5000 dilution) for 2 h. Membranes were washed again before visualization. The Fdbio‐Dura ECL Kit (Fudebio‐tech, China) was used for protein band visualization in photographic films (Biostep, Germany). The following antibodies were used for western blot analysis: p‐Nrf2 (Ab76026, Abcam), NQO1(Ab80588, Abcam), HO‐1(Ab68477, Abcam), BAX (Ab32503, Abcam), Bcl‐2 (Ab182858, Abcam), TLR4 (AF7017, Affinity), MyD88 (AF5195, Affinity), IL‐1β (AF5103, Affinity), IL‐6 (DF6087, Affinity), β‐actin (AF7018, Affinity), goat anti‐rabbit IgG (H+L) HRP (S0001, Affinity) were used as secondary antibodies.

### Diabetic Wound Healing Evaluation

The C57BL/6J mice (6–8 weeks, male) were purchased from HFK Bioscience Co., LTD (Beijing). The C57BL/6 mice were fasted for 12 h and then intraperitoneally injected with streptozotocin (150 mg kg^−1^ in citric acid‐sodium citrate buffer, pH 4.2–4.5) to induce diabetic models. After 1 week, the mice with non‐fasting blood glucose exceeding 16.7 mmol L^−1^ were considered to be diabetic mice. Diabetic mice were anesthetized by intraperitoneal injection of 2% pentobarbital sodium (50 mg kg^−1^), and a disposable biopsy punch was used to create a full‐thickness wound on the back of the mice. The wounds were then treated with phosphate‐buffered saline (PBS), TDNPs, AG, or TAG. The same treatments were applied on day 5. To monitor the wound area, digital photographs were taken on days 1, 4, 7, 10, and 13, respectively. The wound area was quantified using Image J software, and the wound healing rates were calculated as the following formulation: (wound area on day 0 − wound area on day X)/wound area on day 0 × 100%. The mice were sacrificed on day 14, and tissue samples were collected.

### Histology Analysis

Tissue specimens were fixed in 10% formalin and embedded in paraffin. Tissue samples were cut at 5 µm thicknesses, and then sections were deparaffinized and rehydrated, followed by hematoxylin and eosin (H&E) and Masson's trichrome staining. Stained sections were imaged under a microscope. Immunohistochemical staining was performed according to the standard protocols. Sections were incubated with the following antibodies: p‐Nrf2 (Ab76026, Abcam), HO‐1 (Ab52947, Abcam), NQO1 (Ab28947, Abcam), IL‐1β (AF5103, Affinity), IL‐6 (AF6087, Affinity), Ki67 (AF0198, Affinity), CD31 (AF6191, Affinity), and collagen I (AF7001, Affinity).

### Immunofluorescence

L929 cells were fixed with 4% paraformaldehyde for 20 min at 25 °C, washed three times with PBS, permeabilized with 0.2% triton‐X100, and blocked with a PBS‐based solution containing 5% normal goat serum. The tyramide signal amplification (TSA) technology was applied for multiplex immunofluorescence (mIF) staining. In brief, cell slides were incubated with the primary antibody TLR4 (19811‐1‐AP, Proteintech) overnight at 4 °C, followed by treatment with HRP‐conjugated goat anti‐rabbit antibody (5220‐0336, SeraCare) for an additional 50 min at 25 °C. Fluorescent precipitates were generated by treating the slides with the tyramine salt‐AF488 reagent for 20 min at 25 °C via the HRP substrate reaction. Subsequently, the first round of antibodies was removed, and the second round of reactions involving the MyD88 antibody (AF5195, Affinity) (1:200) was initiated following the above‐described procedure. Nuclei were counterstained with DAPI.

Tissues from the wound regions were retrieved after treatment. Tissue was frozen in optimum cutting temperature compound (SAKURA) and then sliced into 5 µm thick sections at −20 °C. The sections were blocked at −20 °C for 1 h with 5% BSA in PBS. Slides were incubated at 4 °C overnight with the primary antibodies. After several washes, sections were incubated with fluorochrome‐conjugated secondary antibodies and DAPI for 60 min. Images were acquired using a confocal microscope and analyzed using ImageJ software (National Institutes of Health).

### ELISA

ELISA kits of mouse IL‐1β ELISA (GEM0002) and mouse IL‐6 ELISA (GEM0001) were obtained from Servicebio biosciences, and mouse IL‐10 ELISA (MAN0017316) was obtained from Thermo Fisher. The ELISA was performed according to the manufacturer's instructions.

### Statistical Analysis

Data were shown mean ± SD. Data preprocessing and sample size (*n*) for each analysis were stated in the relevant figure legends. For normally distributed data, independent two‐sample *t*‐tests were used between two groups; otherwise, the Mann–Whitney U test was used for analysis. To compare data from two or more groups, one‐way analysis of variance (ANOVA) with Tukey's post‐hoc analysis was used for normally distributed data. Otherwise, the non‐parametric Wilcoxon rank‐sum test was used to assess differences (the Kruskal–Wallis test was applied for group tests). All analyses were performed using GraphPad Prism 9.0 and Origin2019b. *P*‐values < 0.05 indicate statistical significance. ns in the graphs indicates no significance; * *p* < 0.05, ** *p* < 0.01, *** *p* < 0.001, and **** *p* < 0.0001.

## Conflict of Interest

The authors declare no conflict of interest.

## Author Contributions

B.W., W.P., and S.L. contributed equally to this work. Z.L., L.B., Z.M.C., W.B.D., and P.W.L. conceptualized the study and managed project administration; W.B.D., P.W.L., L.S.H., X.Q., and Z.M.Z. designed the methodology; W.B.D., L.S.H., L.X.R., and Z.Y.T. performed the investigations; P.W.L., L.S.H., X.Q., Z.Y.T., L.X.R., W.Z.X., L.T., and L.S. visualized the data; L.N.C., N.C.T., L.C.C., and Y.G.H conducted formal analysis; W.B.D., P.W.L., L.S.H. wrote the original draft of the manuscript; Z.L., L.B., and Z.M.C. performed supervision and reviewed the manuscript.

## Supporting information

Supporting Information

Supplemental Video 1

Supplemental Video 2

## Data Availability

The data that support the findings of this study are available from the corresponding author upon reasonable request.
