# Supplementary material for: Turmeric‐Derived Nanoparticles Functionalized Aerogel Regulates Multicellular Networks to Promote Diabetic Wound Healing
Source: Adv Sci (Weinh). 2024 Mar 5;11(18):2307630. doi: 10.1002/advs.202307630 (PMC11095230; doi:10.1002/advs.202307630)
Supplement: Supplementary file 1 — Supporting Information [file ADVS-11-2307630-s002.pdf]

ADVANCED SCIENCE
Open Access

Supporting Information

for *Adv. Sci.*, DOI 10.1002/advs.202307630

Turmeric-Derived Nanoparticles Functionalized Aerogel Regulates Multicellular Networks to Promote Diabetic Wound Healing

Bodeng Wu, Weilun Pan, Shihua Luo, Xiangrong Luo, Yitao Zhao, Qi Xiu, Mingzhen Zhong, Zhenxun Wang, Tong Liao, Ningcen Li, Chunchen Liu, Chengtao Nie, Guanghui Yi, Shan Lin, MengChen Zou, Bo Li* and Lei Zheng**

Supporting Information

Turmeric-Derived Nanoparticles Functionalized Aerogel Regulates Multicellular Networks to Promote Diabetic Wound Healing

Bodeng Wu#, Weilun Pan#, Shihua Luo#, Xiangrong Luo, Yitao Zhao, Qi Xiu, Mingzhen Zhong, Zhenxun Wang, Tong Liao, Ningcen Li, Chunchen Liu, Chengtao Nie, Guanghui Yi, Shan Lin, MengChen Zou, Bo Li*, and Lei Zheng**

B. Wu, W. Pan, Q. Xiu, M. Zhong, Z. Wang, T. Liao, N. Li, C. Liu, C. Nie, G. Yi, S. Lin, B. Li, L. Zheng
Department of Laboratory Medicine, Nanfang Hospital, Southern Medical University Guangzhou 510515, China

S. Luo
Center for Clinical Laboratory Diagnosis and Research, Affiliated Hospital of Youjiang Medical University for Nationalities, Baise 533000, China
Key Laboratory of Research on Clinical Molecular Diagnosis for High Incidence Diseases in Western Guangxi of Guangxi Higher Education Institutions, Affiliated Hospital of Youjiang Medical University for Nationalities, Baise 533000, China

X. Luo, M. Zou
Department of Endocrinology and Metabolism, Nanfang Hospital, Southern Medical University, Guangzhou 510515, China

Y. Zhao
Department of Joint Surgery and Sports Medicine, The Third Affiliated Hospital of Southern Medical University, Guangzhou 510630, China

*Correspondence
Email: zoumc@smu.edu.cn; nfyylibo2012@smu.edu.cn; nfyyzhenglei@smu.edu.cn

#These authors contributed equally

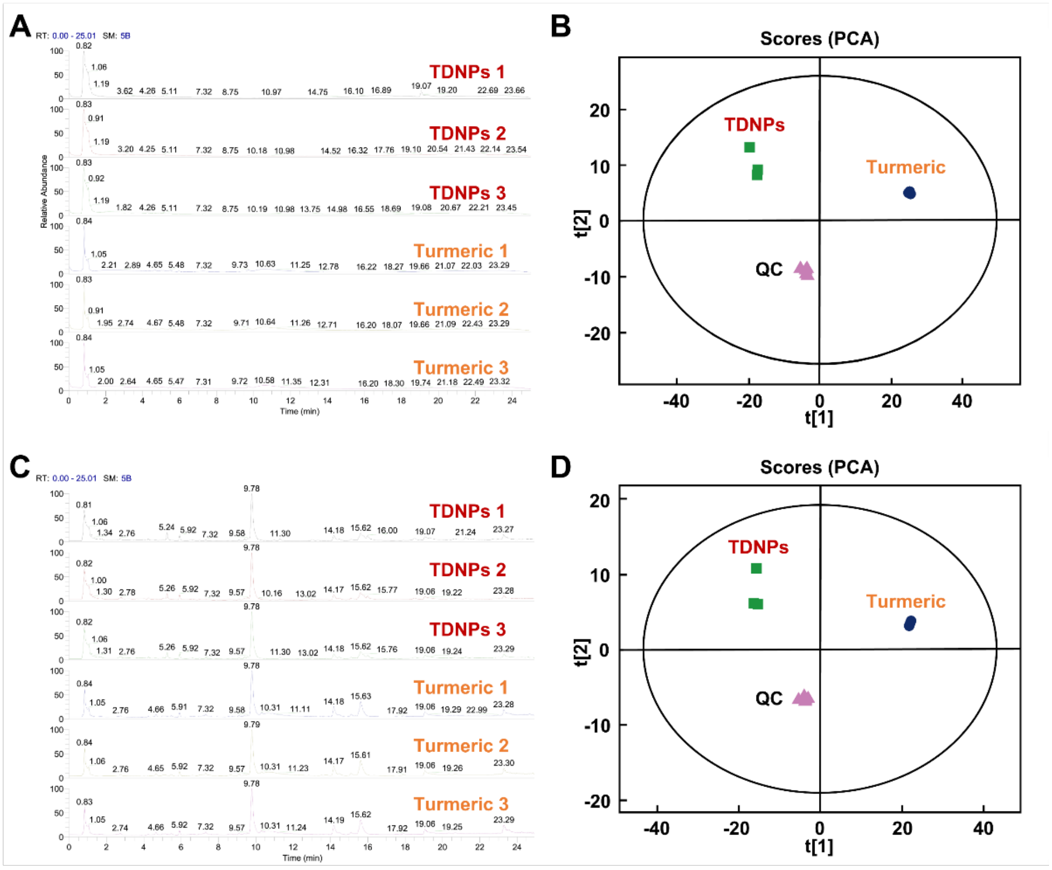

Figure S1. Total ion chromatograms (TICs) and principal component analysis (PCA) of turmeric-derived nanoparticles (TDNPs) and turmeric (*n* = 3). (A) Spectrum of metabolomic negative electrospray ionization (–ESI) modes. (B) PCA of the –ESI modes. (C) Spectrum of metabolomic positive electrospray ionization (+ESI) modes. (D) PCA of the +ESI modes.

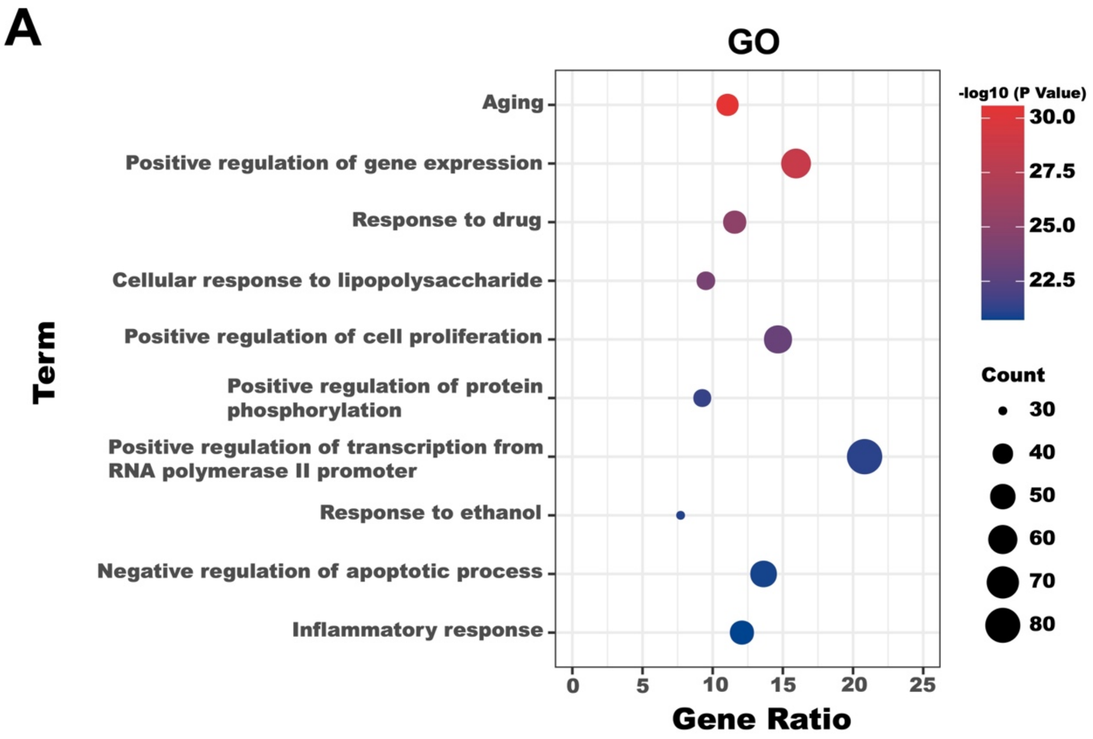

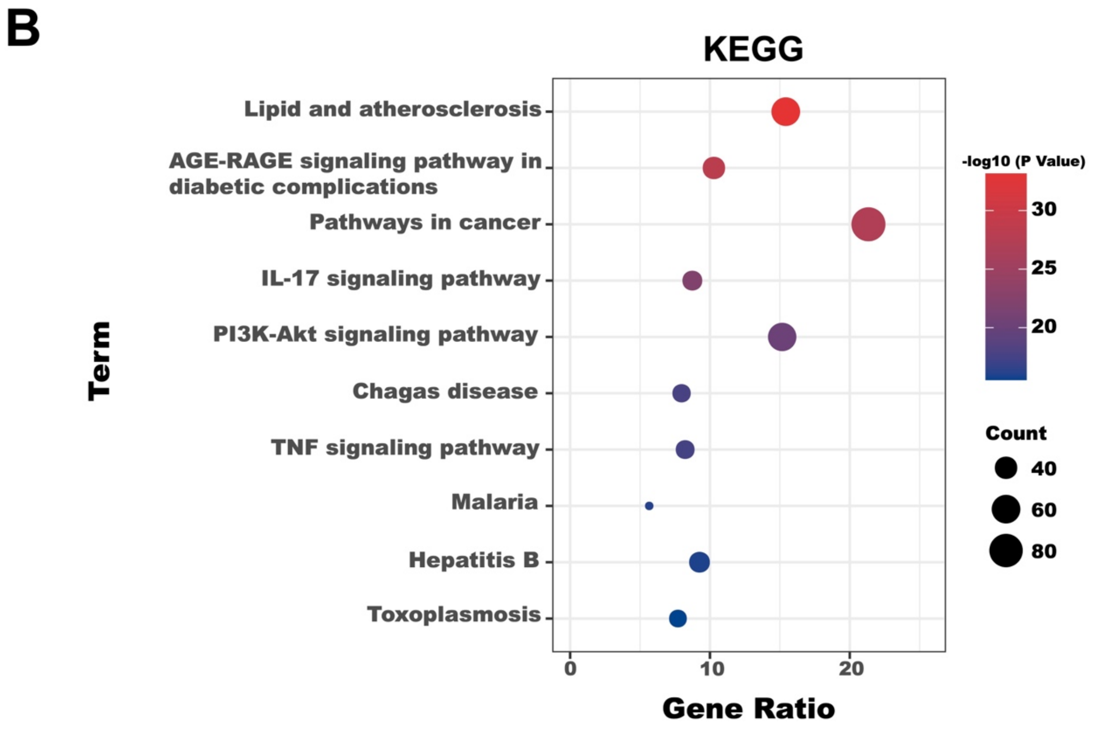

Figure S2. Pathway enrichment analysis of TDNPs target genes. (A) Gene ontology (GO) and (B) Kyoto Encyclopedia of Genes and Genomes (KEGG) enrichment analyses of the TDNPs target genes. TDNPs, turmeric-derived nanoparticles.

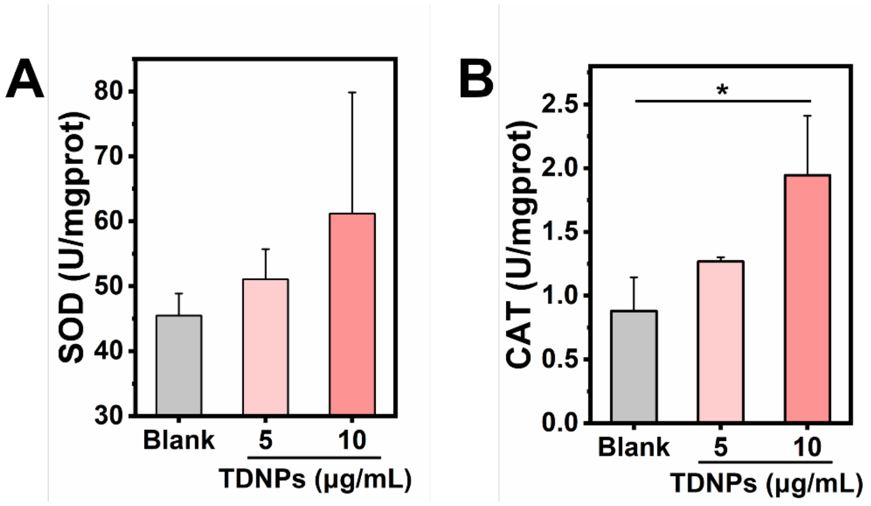

Figure S3. Analysis of antioxidant enzyme activities (n = 3). Activities of the enzymes (A) superoxide dismutase (SOD), and (B) catalase (CAT) in L929 cells. Data are expressed as mean ± SD. Statistical significance was based on one-way ANOVA with post-hoc test; *$p < 0.05$. TDNPs, turmeric-derived nanoparticles.

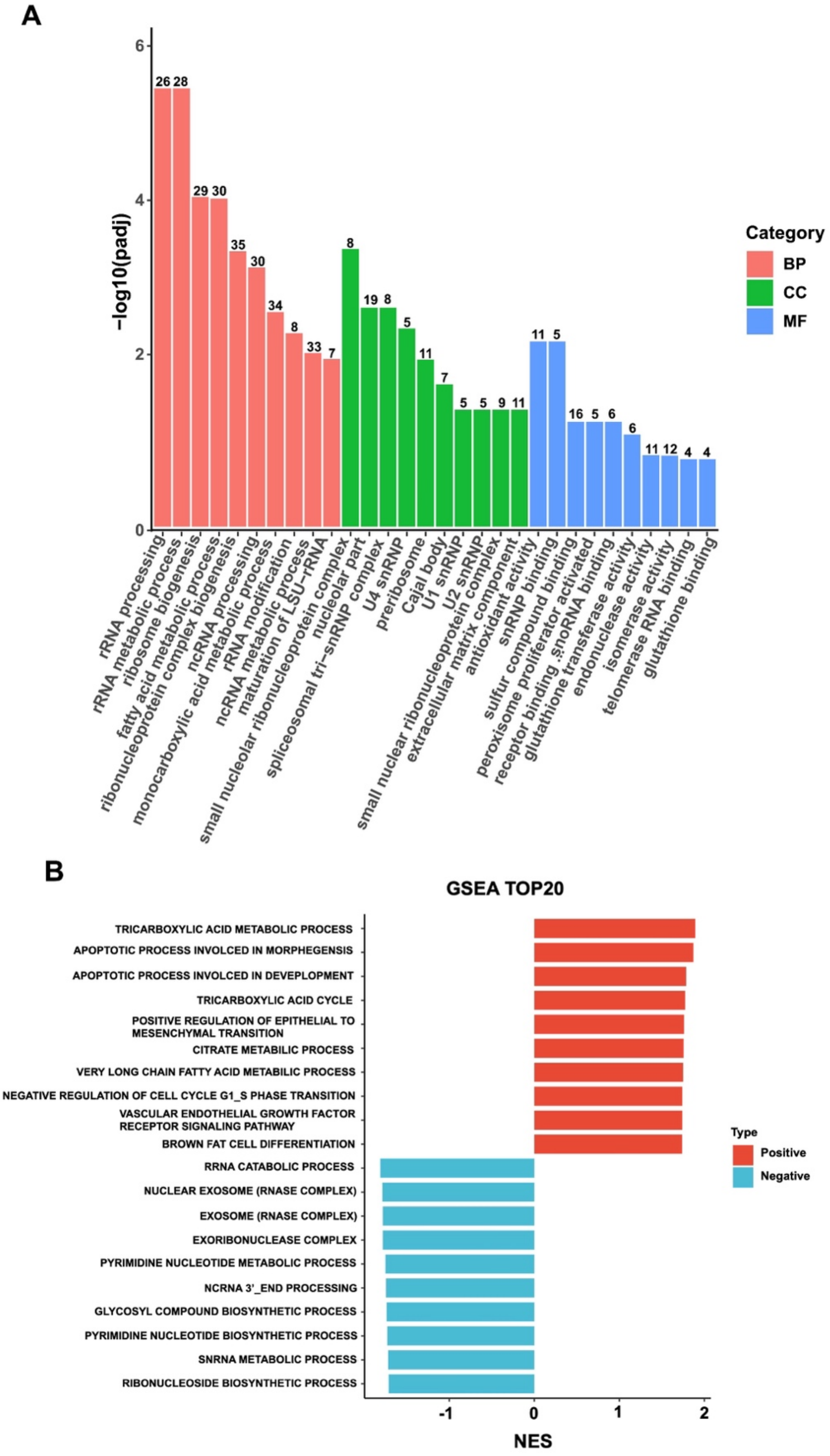

Figure S4. Gene Ontology (GO) and Gene Set Enrichment Analysis (GSEA) of differentially expressed genes (DEGs). (A) Enriched Gene Ontology biological process (BP), cellular component (CC), and molecular function (MF). (B) GSEA pathway enrichment analysis of DEGs.

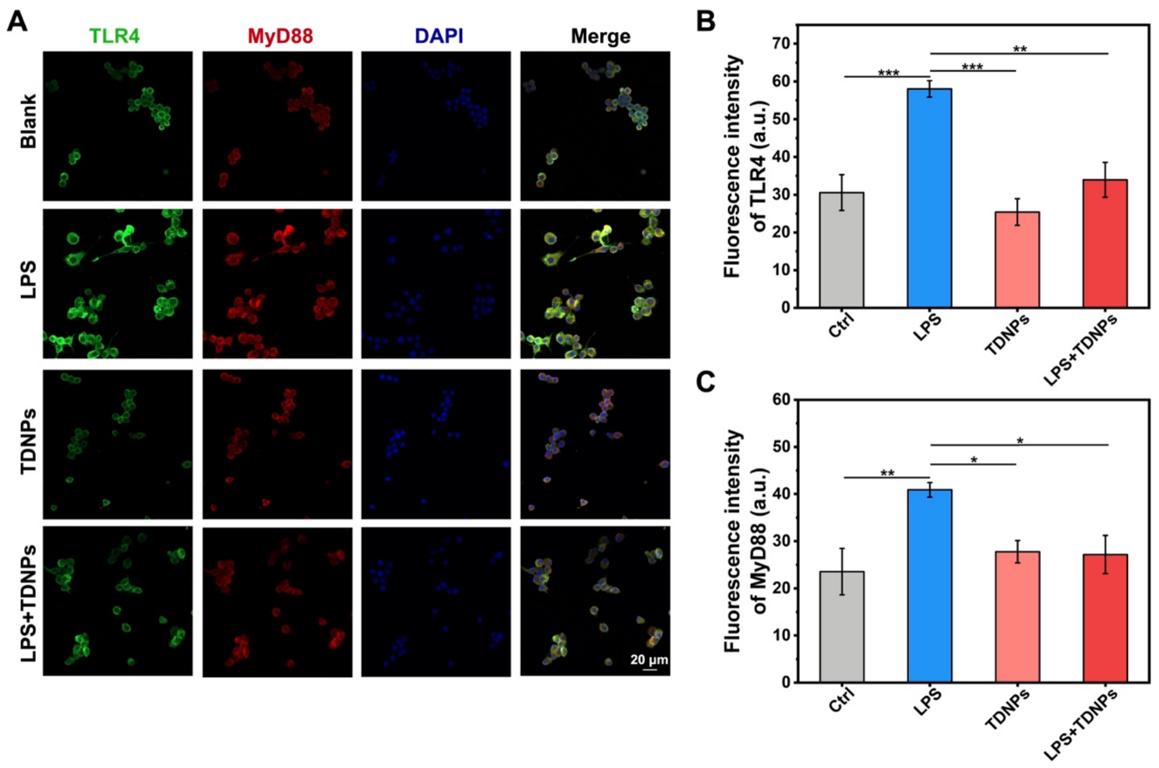

Figure S5. Immunofluorescence analysis of TLR4 and MyD88 expression in macrophages under different treatment conditions. (A) CLSM images of macrophages subjected to various treatments and labeled with TLR4 and MyD88 primary antibodies and secondary fluorescent antibodies. (B, C) Quantification of the (B) TLR4 and (C) MyD88 fluorescence intensities (n = 3). Data are expressed as mean ± SD. Statistical significance was based on one-way ANOVA with post-hoc test; *p < 0.05, **p < 0.01, and ***p < 0.001. CLSM, confocal laser scanning microscopy; LPS, lipopolysaccharide; TDNPs, turmeric-derived nanoparticles.

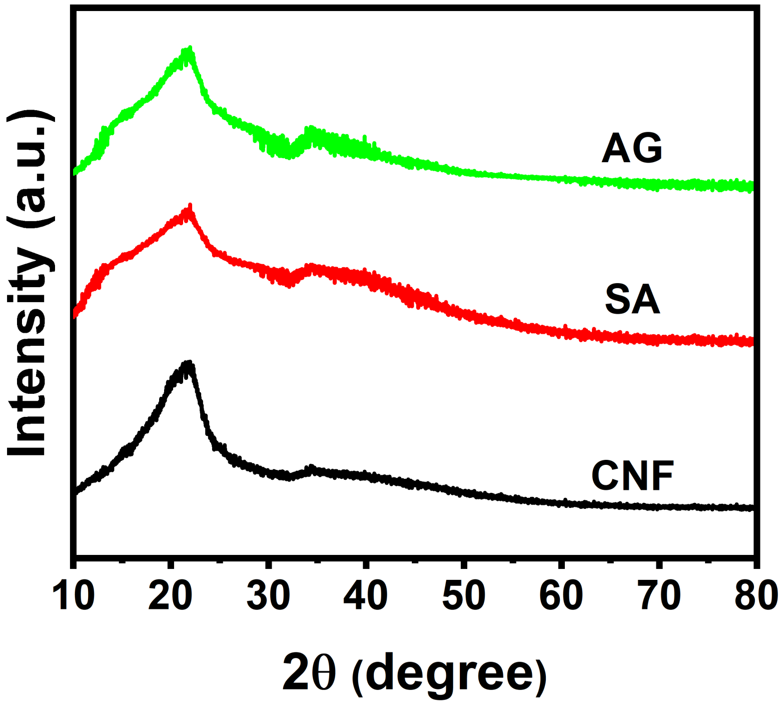

Figure S6. Powder X-ray diffraction (PXRD) patterns of cellulose nanofiber (CNF), sodium alginate (SA), and the aerogel (AG).

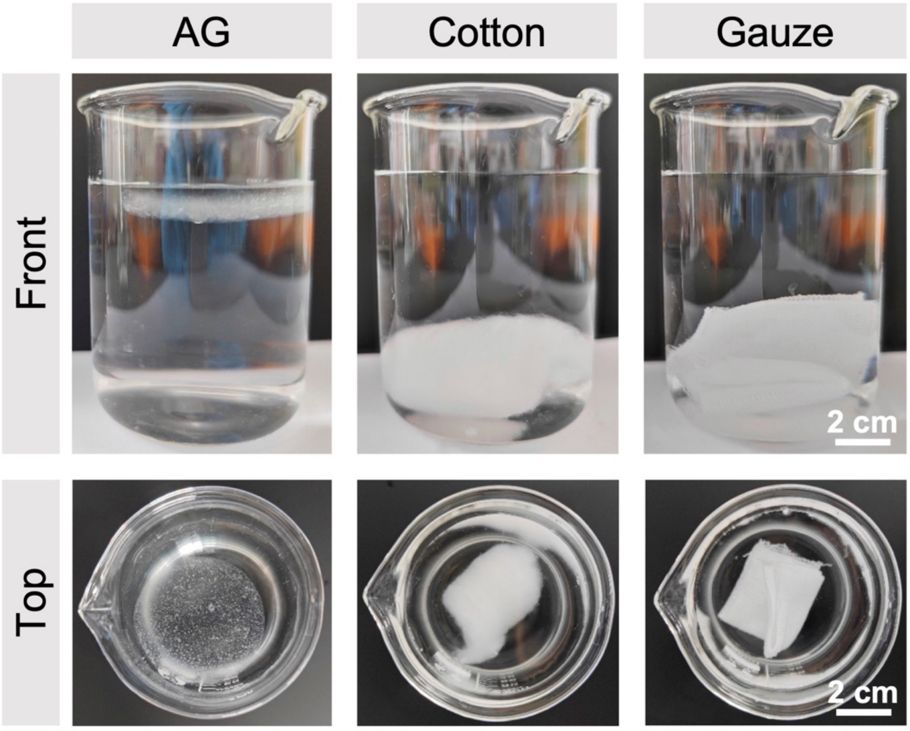

Figure S7. Photographs of the aerogel (AG), medical cotton, and gauze soaked in water.

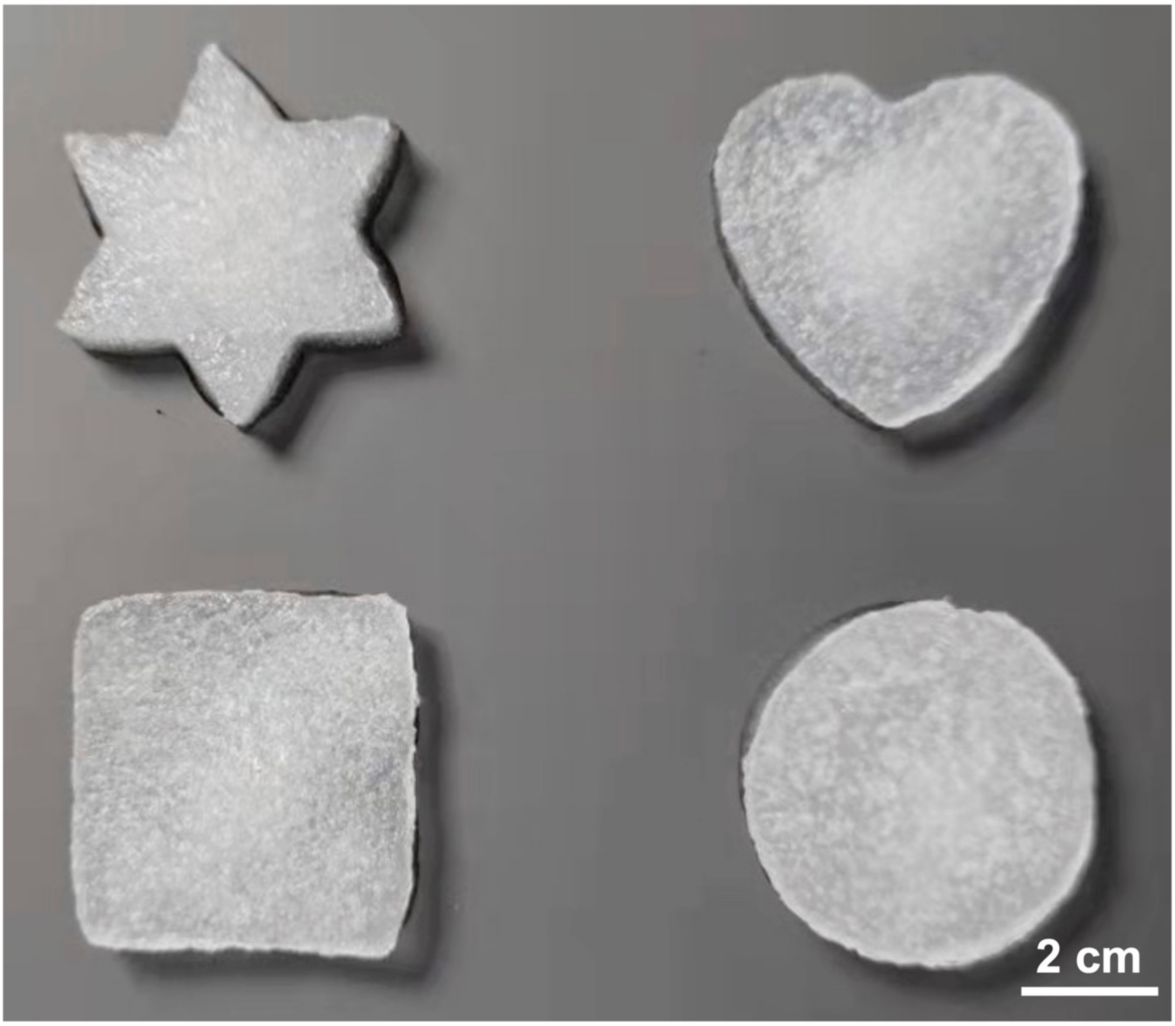

Figure S8. Four pieces of the aerogel (AG) made in different shapes.

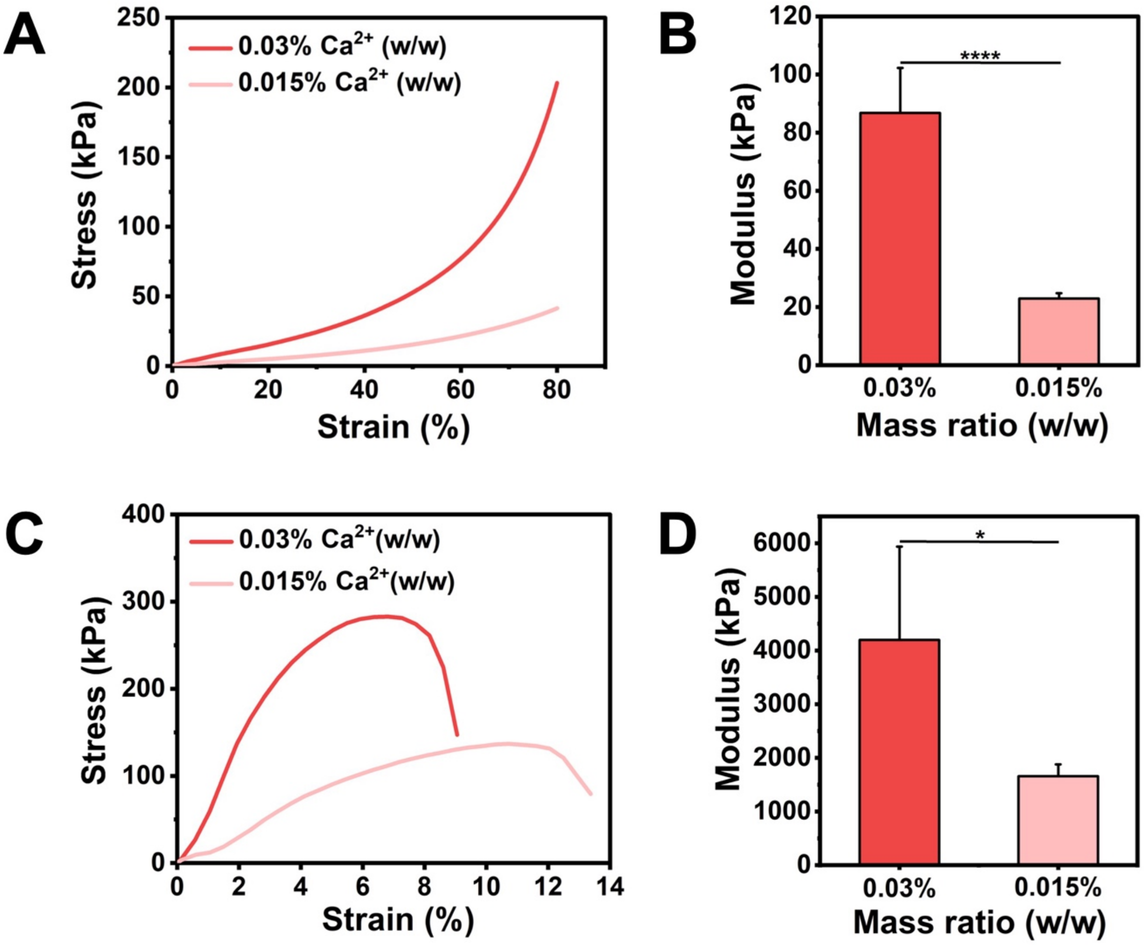

Figure S9. Characterization of the mechanical strength of aerogel (AG) cross-linked with different calcium ion ratios. (A, B) Tensile strength and (C, D) compression performance of AG. Data are shown as mean ± SD. Statistical significance was based on Student's t-test; **p* < 0.05, and *****p* < 0. 0001.

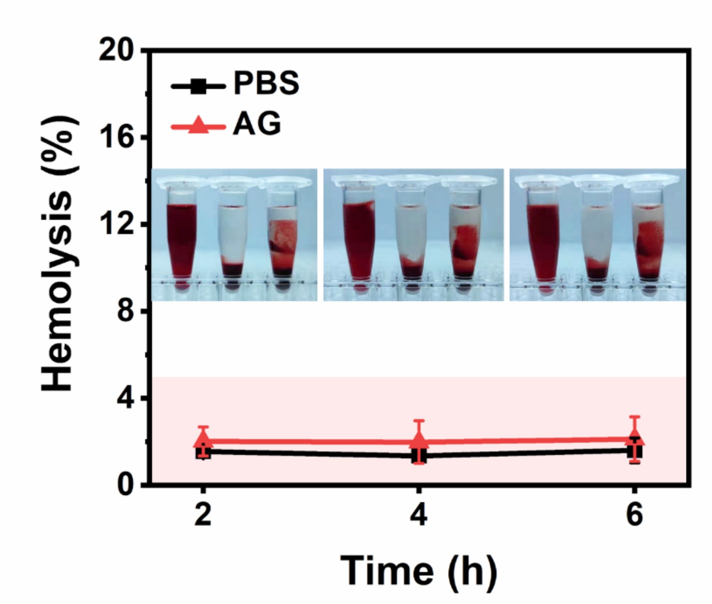

Figure S10. Hemolysis of aerogel (AG). The three tubes in the insert represent (from left to right) the positive control (PBS containing 1% Triton-X), negative control (PBS), and AG-treated red blood cells (n = 3). Data are shown as mean ± SD. PBS, phosphate buffer saline.

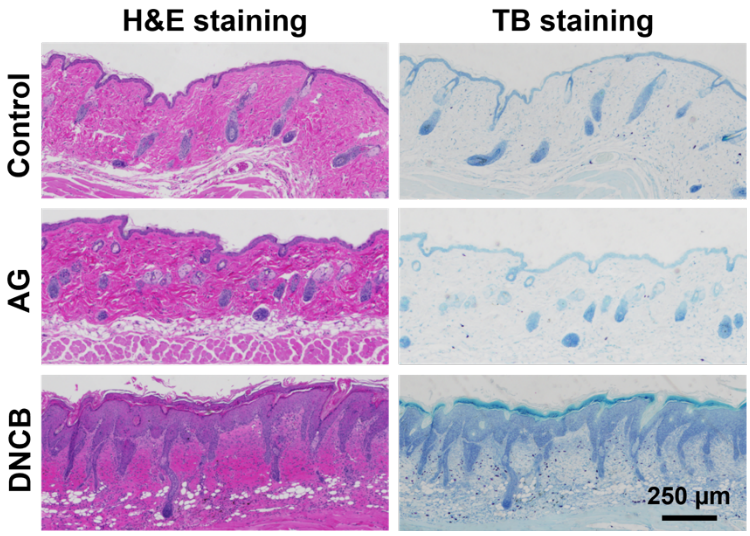

Figure S11. Histological analysis of skin samples collected from mice subjected to different treatments (n = 3). AG, aerogel; DNCB, 1-chloro-2,4-dinitrobenzene; H&E staining, hematoxylin and eosin staining; TB, toluidine blue.

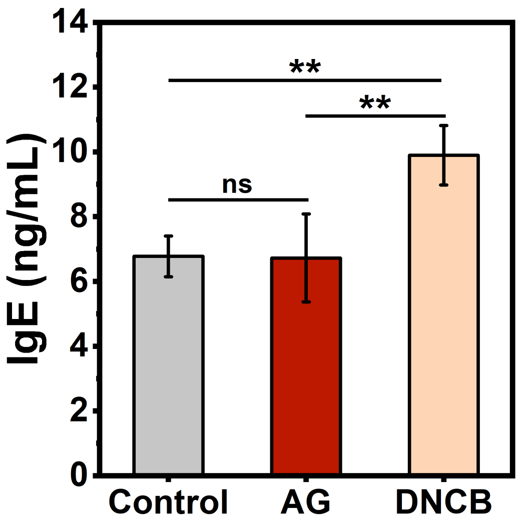

Figure S12. Serum IgE levels in mice with different treatments (*n* = 3). Data are expressed as mean ± SD. Statistical significance was based on one-way ANOVA with post-hoc test; Ns, not significant, $^{**}p < 0.\ 01$. AG, aerogel; DNCB, 1-chloro-2,4-dinitrobenzene.

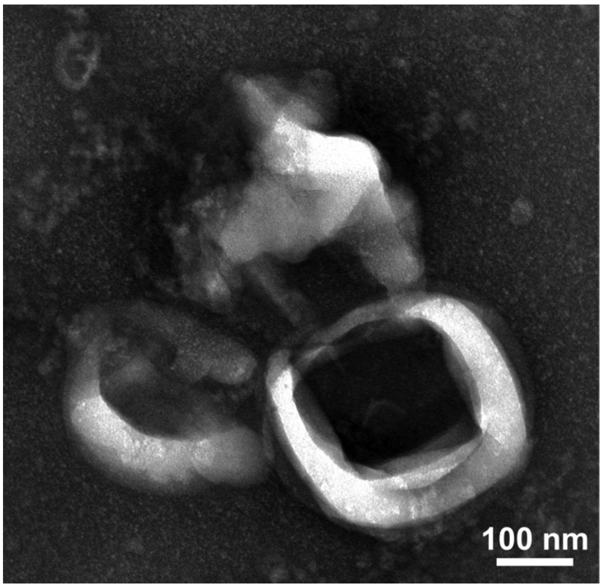

Figure S13. TEM image of TDNPs in TAG on day 49 of storage at 25°C. TEM, transmission electron microscopy; TDNPs, turmeric-derived nanoparticles; TAG, TDNPs-loaded aerogel (TDNPs@AG).

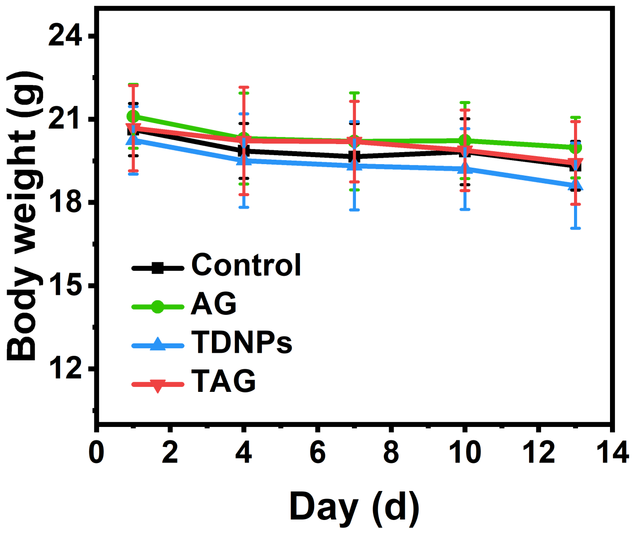

Figure S14. Changes in body weight in different treatment groups ($n \geq 7$). Data are expressed as mean ± SD. Statistical significance was based on one-way ANOVA with post-hoc test. AG, aerogel; TDNPs, turmeric-derived nanoparticles; TAG, TDNPs-loaded aerogel (TDNPs@AG).

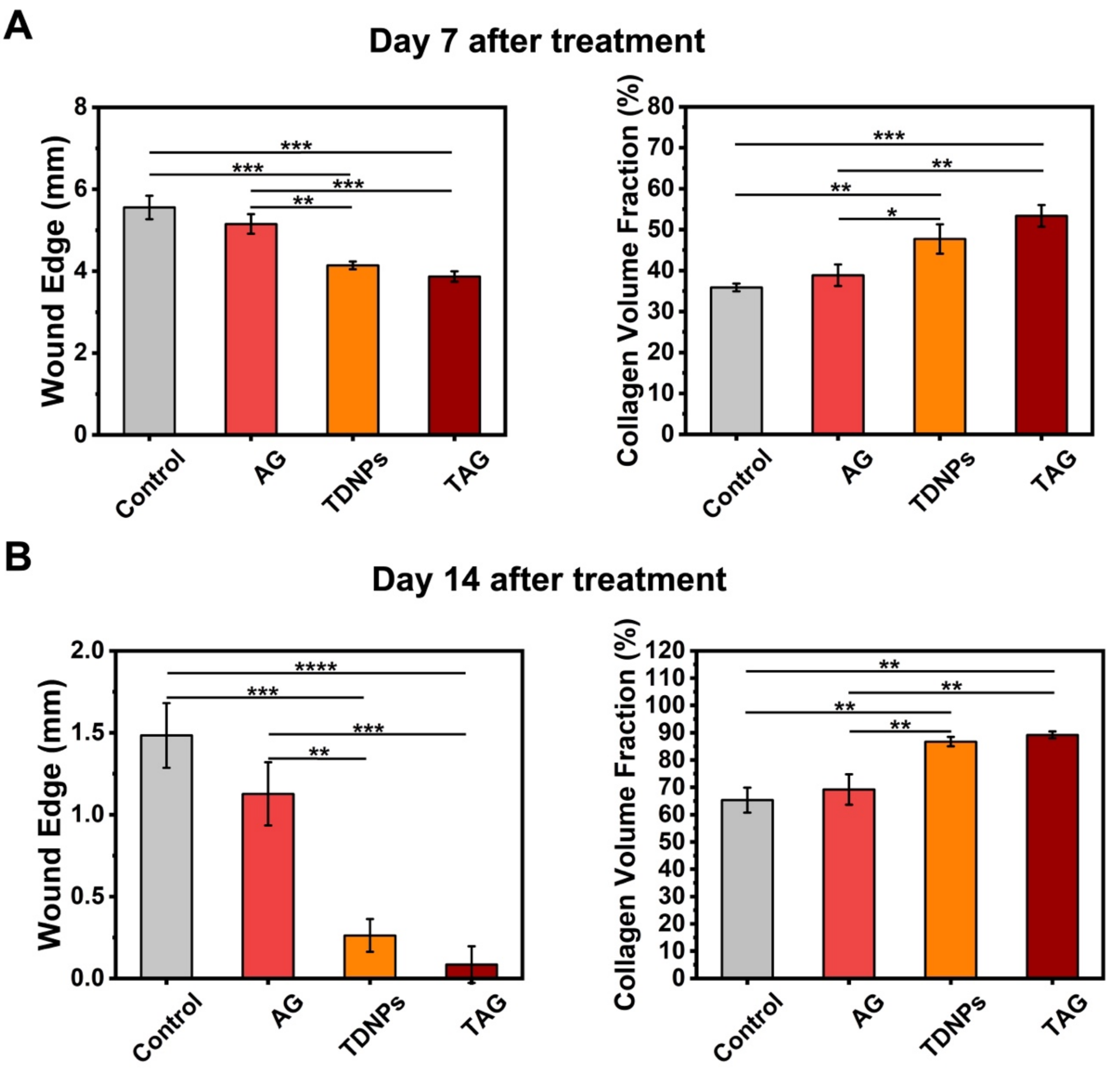

Figure S15. Quantification of (A) wound edge and (B) collagen volume fraction on days 7 and 14 after treatment ($n = 3$). Data are expressed as mean ± SD. Statistical significance was based on one-way ANOVA with post-hoc test; *$p < 0.05$, **$p < 0.01$, ***$p < 0.001$, and ****$p < 0.0001$. AG, aerogel; TDNPs, turmeric-derived nanoparticles; TAG, TDNPs-loaded aerogel (TDNPs@AG).

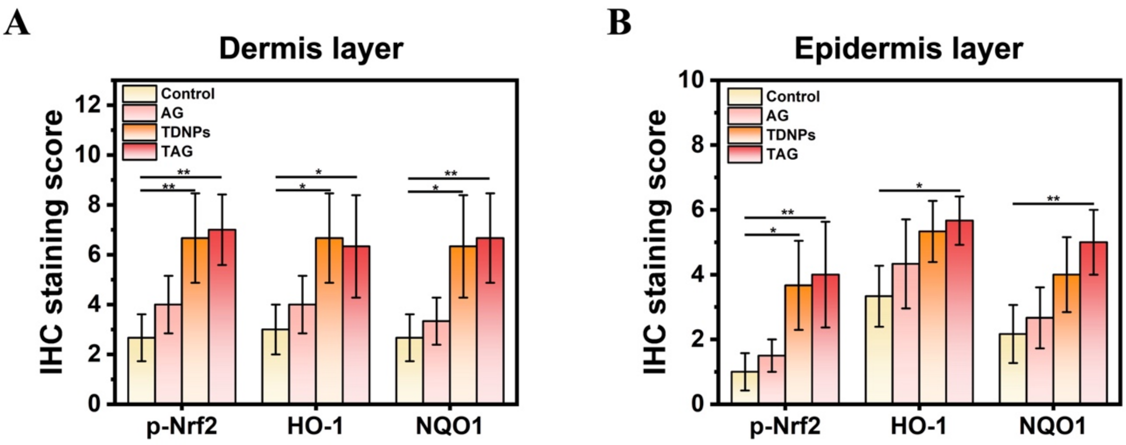

Figure S16. Quantification of immunohistochemical staining scores for p-Nrf2, HO-1, and NQO1 (shown in Figure 8H) (n = 6). Data are expressed as mean ± SD. Statistical significance was based on one-way ANOVA with post hoc test and non-parametric Wilcoxon rank-sum test; *$p < 0.05$ and **$p < 0.01$. AG, aerogel; TDNPs, turmeric-derived nanoparticles; TAG, TDNPs-loaded aerogel (TDNPs@AG).

Table S1. Metabolite composition of turmeric and turmeric-derived nanoparticles (TDNPs).

Name	m/z	rt(s)	Superclass	Proportion of Turmeric	Proportion of TNDPs
Choline	104.1064	49.48	Organic nitrogen compounds	1.22%	15.09%
Polygodial	217.1603	587.549	Undefined	9.10%	14.68%
Ar-turmerone	217.1607	850.568	Lipids and lipid-like molecules	8.17%	8.83%
Retinene	119.085	850.493	Lipids and lipid-like molecules	6.70%	7.61%
Citrate	191.0201	52.83955	Organic acids and derivatives	0.19%	7.03%
Gamma-Nonalactone	121.1009	936.71	Organoheterocyclic compounds	5.27%	4.31%
Farnesal	221.1921	906.161	Lipids and lipid-like molecules	4.03%	3.69%
Curcumenol	235.1722	559.692	Lipids and lipid-like molecules	0.89%	2.78%
Indolelactic acid	188.0714	165.457	Organoheterocyclic compounds	1.30%	2.42%
Erucamide	338.346	1417.735	Undefined	3.14%	2.17%
Phenylalanine	166.0864	104.9815	Organic acids and derivatives	0.92%	2.08%
L-Tryptophan	205.0988	165.587	Organoheterocyclic compounds	0.91%	1.67%
Curcumin	369.1413	662.5945	Phenylpropanoids and polyketides	27.47%	1.30%
N-acetyl-l-phenylalanine	120.0798	104.927	Organic acids and derivatives	0.67%	1.30%
Confertifoline	235.1713	396.985	Undefined	0.63%	1.28%
Adenosine	268.1088	65.30935	Nucleosides, nucleotides, and analogues	0.24%	1.17%
Betaine	118.0843	495.1585	Organic acids and derivatives	0.80%	0.96%
Phthalic acid Mono-2-ethylhexyl Ester	279.1615	913.683	Undefined	0.39%	0.83%
Alpha-Bisabolol	205.1939	897.841	Lipids and lipid-like molecules	0.57%	0.80%
Zerumbone	201.1636	868.368	Lipids and lipid-like molecules	0.48%	0.74%
Etodolac	242.1766	438.971	Organoheterocyclic compounds	0.72%	0.73%
Kinoprene	203.1794	844.3575	Lipids and lipid-like molecules	0.62%	0.71%
Xanthorrhizol	135.0804	389.481	Lipids and lipid-like molecules	0.29%	0.61%
Menthofuran	151.1113	472.678	Lipids and lipid-like molecules	0.17%	0.58%
Tetraethylene glycol	133.0854	315.2625	Organic oxygen compounds	0.01%	0.57%
1,2-Benzenedicarboxylic acid	149.0261	189.006	Undefined	0.67%	0.54%
DL-tryptophan	205.098	158.972	Organoheterocyclic compounds	0.40%	0.53%
Zinniol	265.1462	777.681	Benzenoids	0.01%	0.49%
Sucrose	381.0811	50.731	Organic oxygen compounds	2.08%	0.48%
Cis-aconitate	173.0099	64.97545	Organic acids and derivatives	0.04%	0.46%
Glucose	179.0558	49.61565	Organic oxygen compounds	0.63%	0.41%
Caprylic acid	167.1071	586.421	Lipids and lipid-like molecules	0.21%	0.40%
Perillaldehyde	133.0997	586.425	Lipids and lipid-like molecules	0.20%	0.39%
Fumarate	115.0035	64.2241	Organic acids and derivatives	0.04%	0.34%
Coniferyl alcohol	163.0762	870.389	Benzenoids	0.29%	0.34%
DL-Norvaline	118.0872	63.81645	Undefined	0.59%	0.34%
Pantothenate	220.1147	115.393	Organic oxygen compounds	0.02%	0.31%
4-hydroxybenzoylcholine	224.1049	65.0257	Benzenoids	0.03%	0.30%
Isoalantolactone	215.1442	311.1215	Lipids and lipid-like molecules	0.17%	0.29%
(2-mercaptopropionylamino)acetic acid	146.0294	43.5525	Organic acids and derivatives	0.10%	0.27%
(–)-caryophyllene oxide	203.1772	906.281	Lipids and lipid-like molecules	0.28%	0.27%
Quinate	129.0688	1113.16	Organic oxygen compounds	0.03%	0.26%
Alpha-Cyperone	201.1629	936.71	Lipids and lipid-like molecules	0.26%	0.26%
Succinate	117.0168	73.7976	Organic acids and derivatives	0.02%	0.23%
Diethyltoluamide	192.1391	475.816	Benzenoids	0.24%	0.22%
Demethoxycurcumin	337.1145	620.743	Undefined	9.90%	0.22%

DL-Glutamic acid	148.0611	52.9233	Organic acids and derivatives	0.21%	0.22%
Dioctyl phthalate	391.2857	1376.96	Benzenoids	0.08%	0.21%
2-methyl-3-hydroxybutyric acid	119.0849	255.697	Lipids and lipid-like molecules	0.12%	0.20%
Sclareolide	191.1793	867.9635	Undefined	0.13%	0.20%
Triethylene glycol monobutyl ether	151.0963	325.2645	Organic oxygen compounds	0.00%	0.19%
Farnesol	121.0999	655.373	Lipids and lipid-like molecules	0.12%	0.18%
Tefluthrin	177.0226	52.62045	Benzenoids	0.02%	0.18%
Agomelatine	244.1536	161.221	Organic acids and derivatives	0.08%	0.17%
Ketamine	238.1179	128.874	Benzenoids	0.00%	0.16%
Dibutyl phthalate	279.161	3.03449	Benzenoids	0.12%	0.16%
3,4-dihydroxymandelic acid	149.0225	2.37308	Benzenoids	0.17%	0.16%
Isoeugenyl acetate	207.1229	1339.91	Undefined	0.12%	0.16%
Thiamine monophosphate	122.0694	1495.865	Organoheterocyclic compounds	0.12%	0.16%
Zearalenone	301.1425	911.6615	Phenylpropanoids and polyketides	0.07%	0.16%
Edaravone	175.1113	311.0585	Organoheterocyclic compounds	0.08%	0.16%
Thiosulfuric acid	134.8945	1407.17	Homogeneous non-metal compounds	0.10%	0.15%
C17-sphinganine	288.2914	636.322	Organic nitrogen compounds	0.07%	0.15%
6-benzylaminopurine	225.9901	43.316	Organoheterocyclic compounds	0.02%	0.15%
ProstaglandinF2α	353.2144	994.445	Lipids and lipid-like molecules	0.11%	0.14%
Diisodecyl phthalate	469.3251	1144.09	Benzenoids	0.10%	0.12%
Psoralidin	337.1083	53.5893	Phenylpropanoids and polyketides	0.02%	0.12%
Eflornithine	166.0544	72.6176	Organic acids and derivatives	0.03%	0.12%
DL-asparagine	131.0451	48.1101	Organic acids and derivatives	0.72%	0.11%
Tyr–Ser	269.1157	72.687	Undefined	0.02%	0.11%
4-hydroxy-4′-chlorobiphenyl	203.0176	64.3363	Benzenoids	0.03%	0.11%
Indole-3-carboxaldehyde	146.0594	165.457	Organoheterocyclic compounds	0.05%	0.11%
Azelaic acid	135.0803	640.4865	Lipids and lipid-like molecules	0.21%	0.11%
1,2-propanediol, 3-(1,3-benzodioxol-5-yl)-	195.0506	50.3007	Undefined	0.03%	0.11%
Homocitrate	205.0288	275.277	Organic acids and derivatives	0.00%	0.11%
Parthenolide	249.148	392.778	Lipids and lipid-like molecules	0.09%	0.11%
Citraconic acid	129.0175	65.13515	Lipids and lipid-like molecules	0.01%	0.10%
3-furancarboxylic acid, tetrahydro-4-methylene-5-oxo-2-propyl-, (2r,3s)-rel-	183.065	256.752	Organoheterocyclic compounds	0.07%	0.10%
Bisphenol a	229.1432	315.2625	Benzenoids	0.00%	0.10%
Cuminyl alcohol	105.069	586.115	Lipids and lipid-like molecules	0.07%	0.10%
Cis-jasmone	107.0841	590.392	Organic oxygen compounds	0.03%	0.09%
4-pyridinecarboxylic acid	124.0388	44.2637	Organoheterocyclic compounds	0.09%	0.09%
2-butanol, 1-[(2,3-dihydro-7-methyl-1h-inden-4-yl)oxy]-3-[(1-methylethyl)amino]-, (2r,3r)-rel-	260.1865	209.711	Undefined	0.03%	0.09%
Octadecanoic acid	283.2633	1365.655	Lipids and lipid-like molecules	0.02%	0.09%
trans-3-Coumaric acid	147.0468	405.1165	Undefined	0.11%	0.09%
Beta-Damascone	137.0956	868.422	Organic oxygen compounds	0.05%	0.08%
Moroxydine	172.1337	55.1237	Organic nitrogen compounds	0.00%	0.08%
Trans-ferulic acid	177.0532	432.462	Phenylpropanoids and polyketides	0.34%	0.08%
(+/–)-n-acetyl-3,4-methylenedioxyamphetamine	222.1261	239.079	Undefined	0.02%	0.07%
Myo-inositol	127.0377	128.843	Organic oxygen compounds	0.07%	0.07%
Clozapine	327.1582	800.7975	Organoheterocyclic compounds	0.04%	0.07%
4-(methylnitrosamino)-1-(3-pyridyl)-1-butanol	192.1223	1457.06	Undefined	0.06%	0.07%
D-aspartic acid	132.031	61.88995	Organic acids and derivatives	0.06%	0.07%
Glutaric acid	113.0102	70.0025	Organic acids and derivatives	0.03%	0.07%
Germacrone	219.1695	338.116	Lipids and lipid-like molecules	0.03%	0.06%

Phenylacetaldehyde	103.0534	105.278	Benzenoids	0.02%	0.06%
L-Glutamate	130.0514	49.0098	Organic acids and derivatives	0.11%	0.06%
Sempervirine	273.1466	390.0535	Organoheterocyclic compounds	0.07%	0.06%
Gamma-Linolenoyl ethanolamide	322.2734	1057.38	Organic nitrogen compounds	0.04%	0.06%
3-ureidopropionic acid	155.0392	49.9976	Organic acids and derivatives	0.05%	0.06%
1-(1′,3′-benzodioxol-5′-yl)-2-butanamine	194.1204	585.606	Undefined	0.04%	0.06%
1,3-benzenediol, 5-methyl-4-[(1r,6r)-3-methyl-6-(1-methylethenyl)-2-cyclohexen-1-yl]-	259.1652	655.904	Lipids and lipid-like molecules	0.04%	0.06%
Chamazulene	157.1015	587.486	Lipids and lipid-like molecules	0.03%	0.06%
Camphor	153.1091	1445.37	Lipids and lipid-like molecules	0.05%	0.06%
His–Lys	284.0992	72.4559	Undefined	0.01%	0.05%
Prednisolone	181.1021	946.32	Lipids and lipid-like molecules	0.00%	0.05%
Docosanamide	340.3562	1496.23	Lipids and lipid-like molecules	0.07%	0.05%
3-cyclohexyl-1,1-dimethylurea	171.1388	568.466	Organic acids and derivatives	0.04%	0.05%
N8-acetylspermidine	171.1383	1349.44	Organic acids and derivatives	0.05%	0.05%
Oxycodone	316.1748	155.0975	Undefined	0.00%	0.05%
Alantolactone	215.1424	415.3275	Lipids and lipid-like molecules	0.01%	0.05%
Hydrocortisone	345.1873	256.321	Lipids and lipid-like molecules	0.01%	0.05%
[6]-gingerol	277.1772	385.064	Benzenoids	0.04%	0.05%
13-keto-9z,11e-octadecadienoic acid	295.2281	871.6	Lipids and lipid-like molecules	0.00%	0.05%
Cocaine	304.1713	58.3935	Benzenoids	0.01%	0.04%
2-aminoadipic acid	144.0656	164.5805	Organic acids and derivatives	0.02%	0.04%
Mesoxalate	116.9827	43.01365	Organic acids and derivatives	0.04%	0.04%
Thiodiglycol sulfoxide	139.0396	77.9915	Undefined	0.01%	0.04%
N-carboxyethyl-gamma-aminobutyric acid	140.0797	1439.55	Organic acids and derivatives	0.05%	0.04%
Isopentenyladenine	204.1231	1455.515	Organoheterocyclic compounds	0.03%	0.04%
Bisdemethoxycurcumin	309.1176	581.71	Undefined	1.07%	0.04%
Ethyl glucuronide	281.093	264.901	Organic oxygen compounds	0.00%	0.04%
Galanthamine	231.0986	1099.435	Undefined	0.01%	0.04%
Desipramine	231.1607	183.344	Organoheterocyclic compounds	0.02%	0.04%
L-hydroxyarginine	128.0612	959.246	Organic acids and derivatives	0.01%	0.03%
L-pyroglutamic acid	128.0351	48.8321	Organic acids and derivatives	0.02%	0.03%
13s-hydroperoxy-9z,11e-octadecadienoic acid	293.2145	870.927	Lipids and lipid-like molecules	0.00%	0.03%
11-(phenylcarbonyl)-7,11-diazatricyclo[7.3.1.0<2,7>]trideca-2,4-dien-6-one	295.1347	678.0765	Organoheterocyclic compounds	0.00%	0.03%
Rotundine	356.19	971.9135	Undefined	0.00%	0.03%
5alpha-androstan-17beta-ol-3-one	173.1298	1220.265	Lipids and lipid-like molecules	0.02%	0.03%
L-dihydroorotate	157.0246	194.71	Organic acids and derivatives	0.01%	0.03%
DL-threonine	102.054	52.16405	Organic acids and derivatives	0.03%	0.03%
Dibutyl adipate	259.1667	553.026	Lipids and lipid-like molecules	0.00%	0.03%
2-hydroxybenzyl alcohol	107.0705	296.8415	Benzenoids	0.02%	0.03%
Gestrinone	309.1688	269.414	Lipids and lipid-like molecules	0.02%	0.03%
L-Histidine	156.0743	59.0566	Organic acids and derivatives	0.22%	0.03%
Oleoyl ethylamide	310.3024	1231.46	Lipids and lipid-like molecules	0.02%	0.03%
L-propionylcarnitine	240.1179	130.245	Lipids and lipid-like molecules	0.00%	0.03%
Thioperamide	293.1721	240.9115	Undefined	0.02%	0.03%
Dodecanoic acid, 12-[[(cyclohexylamino)carbonyl]amino]-	198.1852	439.246	Lipids and lipid-like molecules	0.03%	0.03%
Isopentenyl-adenine-9-glucoside	366.1779	226.824	Organic oxygen compounds	0.01%	0.03%
Pimelic acid	221.0968	279.433	Lipids and lipid-like molecules	0.02%	0.02%
Nootkatone	145.1011	586.179	Lipids and lipid-like molecules	0.02%	0.02%
Cycloate	216.1253	1492.845	Organosulfur compounds	0.02%	0.02%

D-Psicose	127.0384	49.764	Organic oxygen compounds	0.70%	0.02%
Tetrahydrocurcumin	355.1561	297.829	Phenylpropanoids and polyketides	0.06%	0.02%
D-Mannose	163.0562	93.0283	Organic oxygen compounds	0.01%	0.02%
Huperzine b	257.1514	700.33	Undefined	0.03%	0.02%
Matairesinol	376.1785	240.287	Lignans, neolignans, and related compounds	0.00%	0.02%
Metronidazole	128.062	781.903	Organoheterocyclic compounds	0.01%	0.02%
Kendomycin	485.2839	1156.12	Undefined	0.01%	0.02%
N-octadecylamine	270.3167	993.119	Organic nitrogen compounds	0.06%	0.02%
D-glucuronic acid	101.0238	60.35715	Organic oxygen compounds	0.01%	0.02%
16-Hydroxypalmitic acid	271.2313	800.546	Lipids and lipid-like molecules	0.01%	0.02%
(–)-Secoisolariciresinol	361.19	292.9405	Lignans, neolignans and related compounds	0.00%	0.02%
Rac-7-hydroxypropranolol	258.1534	120.75	Benzenoids	0.00%	0.02%
(–)-Perillyl alcohol	107.085	935.649	Lipids and lipid-like molecules	0.01%	0.02%
Cinchonine	293.1808	979.565	Undefined	0.00%	0.02%
5-hydroxymethyl-2-furancarboxylic acid	143.0343	165.4265	Organoheterocyclic compounds	0.01%	0.02%
Dextromethorphan	272.1866	207.322	Undefined	0.01%	0.01%
5-androstene-3β,17β-diol	131.0853	586.3845	Lipids and lipid-like molecules	0.00%	0.01%
Galactinol	387.1164	219.419	Organic oxygen compounds	0.01%	0.01%
(z)-5,8,11-trihydroxyoctadec-9-enoic acid	348.2766	451.5545	Lipids and lipid-like molecules	0.00%	0.01%
P-methoxycinnamic acid ethyl ester	207.1264	308.584	Phenylpropanoids and polyketides	0.05%	0.01%
Diethylstilbestrol	269.1693	244.4565	Undefined	0.01%	0.01%
Trifluoperazine	425.1927	261.5765	Undefined	0.00%	0.01%
3-hydroxyanthranilic acid	136.052	643.12	Benzenoids	0.01%	0.01%
Kynurenic acid	212.0197	405.864	Organoheterocyclic compounds	0.00%	0.01%
Tetraethylene glycol monomethyl ether	103.0536	936.16	Organic oxygen compounds	0.01%	0.01%
Indole	135.0888	953.198	Organoheterocyclic compounds	0.01%	0.01%
Alpha-Piperidinobutiophenone	232.1665	951.693	Organic oxygen compounds	0.01%	0.01%
Alprenolol	250.1793	1238.765	Benzenoids	0.01%	0.01%
Mefloquine	361.1205	442.0255	Undefined	0.02%	0.01%
Pyrrolidine, 2-(diphenylmethyl)-, (2s)-	143.0855	585.767	Benzenoids	0.01%	0.01%
Threitol	123.0802	326.0685	Organic oxygen compounds	0.01%	0.01%
Benzoic acid, 4-[[(5,6,7,8-tetrahydro-5,5,8,8-tetramethyl-2-naphthalenyl)carbonyl]amino]-	215.143	664.464	Benzenoids	0.03%	0.01%
Amastatin	473.2784	1265.95	Organic acids and derivatives	0.00%	0.01%
Diphenylamine	170.0943	49.0199	Benzenoids	0.01%	0.01%
Isonicotinamide	123.0553	419.6985	Organoheterocyclic compounds	0.01%	0.01%
3-phenoxy-1-propanol	135.0808	1052.82	Undefined	0.01%	0.01%
Hydrocotarnine	222.1121	279.418	Undefined	0.01%	0.01%
Sphingosine	300.293	836.694	Organic nitrogen compounds	0.00%	0.01%
N2,N2-Dimethylguanosine	312.133	108.15	Undefined	0.00%	0.01%
DL-serine	104.0346	47.4748	Organic acids and derivatives	0.04%	0.01%
1-naphthol	143.0486	584.623	Benzenoids	0.08%	0.01%
Tris(hydroxymethyl)aminomethane	122.0598	97.82105	Organic nitrogen compounds	0.01%	0.01%
DL-tyrosine	182.0807	51.9498	Organic acids and derivatives	0.40%	0.01%
3′-*O*-methyladenosine	282.116	52.339	Nucleosides, nucleotides, and analogues	0.01%	0.01%
Palmitamide	256.2616	640.6415	Lipids and lipid-like molecules	0.01%	0.01%
Secoisolariciresinol	361.1728	309.281	Lignans, neolignans and related compounds	0.28%	0.01%
4-coumaroylcholine	250.1569	282.9455	Phenylpropanoids and polyketides	0.00%	0.01%
Benzothiazole, 2-methylmercapto-	182.0102	574.171	Undefined	0.00%	0.01%
Trimethoprim	291.1573	419.118	Benzenoids	0.01%	0.01%

Dibenzylamine	198.1283	268.9815	Benzenoids	0.00%	0.01%
3-Phenylpropanoic acid	133.0616	634.75	Phenylpropanoids and polyketides	0.01%	0.00%
Isoxaben	331.1595	434.8695	Benzenoids	0.01%	0.00%
Benzylazanium	107.0746	542.454	Undefined	0.00%	0.00%
Saccharin	181.9928	1488.11	Undefined	0.00%	0.00%
9-hydroperoxy-10e,12z-octadecadienoic acid	293.212	778.622	Lipids and lipid-like molecules	0.00%	0.00%
16-hydroxyhexadecanoic acid	271.2293	825.672	Lipids and lipid-like molecules	0.00%	0.00%
3-hydroxy-3-methylglutaric acid	109.0281	55.5533	Lipids and lipid-like molecules	0.02%	0.00%
Glucose-1-phosphate	241.0112	58.3682	Organic oxygen compounds	0.01%	0.00%
Pentaethylene glycol	239.147	162.897	Organic oxygen compounds	0.00%	0.00%
1,1,1-tris(hydroxymethyl)propane	269.1884	383.255	Organic oxygen compounds	0.01%	0.00%
Stearic acid	283.2685	1405.31	Lipids and lipid-like molecules	0.00%	0.00%
L-Fucose	165.0554	43.0495	Organic oxygen compounds	0.18%	0.00%
Enterodiol	325.1478	506.176	Lignans neolignans and related compounds	0.07%	0.00%
Pro–Trp	302.3027	749.2185	Organic nitrogen compounds	0.01%	0.00%
Benzamide, n-[(1s)-1-(aminocarbonyl)-4-[(2-chloro-1-iminoethyl)amino]butyl]-	311.1322	850.24	Benzenoids	0.00%	0.00%
1-Oleoyl-sn-glycero-3-phosphocholine	522.3442	886.073	Undefined	0.00%	0.00%

Table S2. Number of interactional genes of the 10 active metabolites in Comparative Toxicogenomics Database (CTD).

Name	Inter-gene number
Choline	5030
Ar-turmerone	7
Retinene	58
Citrate	42
Phenylalanine	493
L-Tryptophan	24
Curcumin	982
Adenosine	795
Betaine	227
Zerumbone	61

Table S3. Water vapor transmission rate (WVTR) analysis of the aerogel (AG).

Number	Cup	Weighing frequency	Weight (g)	WVTR [g/(m^2/day)]
1	1	1	121.6894	0.0000
2	1	2	120.9407	2038.4636
3	1	3	120.1983	2021.3107
4	1	4	119.4773	1963.0456
5	1	5	118.7584	1957.3280
6	1	6	118.0500	1928.7399
7	1	7	117.3442	1921.6610
8	1	8	116.6459	1901.2410
9	1	9	115.9500	1894.7065
10	1	10	115.2580	1884.0881
11	1	11	114.5687	1876.7369
12	1	12	113.8803	1874.2865
13	1	13	113.2000	1852.2329
14	1	14	112.5277	1830.4515
1	2	1	126.1204	0.0000
2	2	2	125.2531	2361.3723
3	2	3	124.4006	2321.0768
4	2	4	123.5660	2272.3410
5	2	5	122.7412	2245.6588
6	2	6	121.9244	2223.8774
7	2	7	121.1151	2203.4574
8	2	8	120.3125	2185.2155
9	2	9	119.5163	2167.7904
10	2	10	118.7322	2134.8461
11	2	11	117.9398	2157.4443
12	2	12	117.1611	2120.1437
13	2	13	116.3883	2104.0799

Table S4. Primers used for RT-PCR

Gene	Primer sequence (5′ —3′)
IL-1β	Forward: GAAATGCCACCTTTTGACAGTG<br>Reverse: TGGATGCTCTCATCAGGACAG
TNF-α	Forward: CCCTCACACTCAGATCATCTTCT<br>Reverse: GCTACGACGTGGGCTACAG
INF-γ	Forward: ATGAACGCTACACACTGCATC<br>Reverse: CCATCCTTTTGCCAGTTCCTC
IL-6	Forward: TAGTCCTTCCTACCCCAATTTCC<br>Reverse: TTGGTCCTTAGCCACTCCTTC
IL-10	Forward: CTTACTGACTGGCATGAGGATCA<br>Reverse: GCAGCTCTAGGAGCATGTGG
TGF-β	Forward: CTCCCGTGGCTTCTAGTGC<br>Reverse: GCCTTAGTTTGGACAGGATCTG
COL1A1	Forward: GTGCGATGACGTGATCTGTGA<br>Reverse: CGGTGGTTTCTTGGTCGGT
Fibronectin1	Forward: GAGAATAAGCTGTACCATCGCAA<br>Reverse: CGACCACATAGGAAGTCCCAG
VEGF	Forward: GCACATAGAGAGAATGAGCTTCC<br>Reverse: CTCCGCTCTGAACAAGGCT
β-actin	Forward: ACATCCGTAAAGACCTCTATGCC<br>Reverse: ACCGATCCACACAGAGTACTTGC
